# From Closed to Mighty: How GTP-Induced Allosteric Dynamics, Dimerization, and Membrane Binding Transform a Bacterial Dynamin-like Protein

**DOI:** 10.1021/acs.jcim.6c01952

**Published:** 2026-09-23

**Authors:** Wibke Schumann, Bastian F. Bundschuh, Jennifer Loschwitz, Birgit Strodel

**Affiliations:** † Faculty of Mathematics and Natural Sciences, 9170 Heinrich Heine University Düsseldorf, 40225 Düsseldorf, Germany; ‡ Institute of Biological Information Processing: Structural Biochemistry, 28334 Forschungszentrum Jülich, 52428 Jülich, Germany

## Abstract

Dynamins, crucial GTPases involved in membrane remodeling, play key roles in processes like endocytosis and membrane fission. While their importance is well-established, the precise dynamics of their conformational changes remain unclear. This study investigates the bacterial dynamin-like protein (BDLP) from *Nostoc punctiforme*, which undergoes a dramatic closed-to-open transition upon GTP binding. Using standard and enhanced all-atom molecular dynamics simulations, we map this transition, revealing a complex energetic landscape. The initial barrier arises from disruption of salt bridges formed by the GTPase domain with the trunk and paddle, setting off the main hinge motion. Subsequent conformational changes involve further hinge motions and lateral shearing to minimize electrostatic forces. Notably, GTP binding accelerates the transition by 5 orders of magnitude through an allosteric mechanism. Our multiscale simulations of 37.1 μs accumulated simulation time show that BDLP dimerization and membrane binding are necessary to initiate the stabilization of the open conformation. These findings provide crucial insights into BDLP’s membrane remodeling mechanism, advancing our understanding of dynamin dynamics.

## Graphical abstract

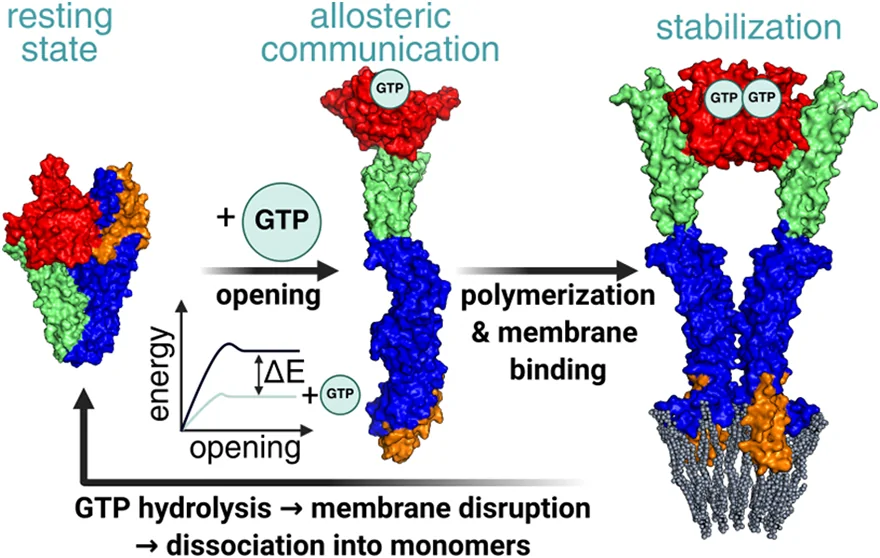

## 1 Introduction

Dynamin-superfamily proteins are a diverse group of GTPase enzymes that play crucial roles in various cellular functions, primarily related to membrane dynamics.^1–4^ Dynamins are remarkable in their mechanochemical capabilities, generating some of the highest torques known for proteins, in the range of a few nN·nm, corresponding to the attojoule range; indeed, these molecular forces are only exceeded by flagellar proteins.^1,5^ Dynamin is most recognized for its essential role in clathrin-mediated endocytosis, where it assembles at the necks of budding vesicles to facilitate membrane fission and release vesicles into the cytoplasm.^6^ Beyond endocytosis, dynamin-like proteins are involved in the fission of various membrane types, including the mitochondrial membrane during division, ensuring proper organelle distribution during cell division.^7,8^ Their membrane-fission capabilities are vital for vesicle trafficking, cytoskeletal interactions that maintain cell shape and promote intracellular movement, and immune responses through the regulation of immune receptor endocytosis.^9^ Additionally, dynamins are hijacked by viruses to facilitate their budding and release from host cells.^10,11^ Conversely, certain members of the dynamin superfamily, particularly those related to synaptic transmission, are implicated in membrane fusion processes, playing key roles in the release of neurotransmitters.^12^

Structurally, dynamin-superfamily proteins contain a conserved GTPase (G) domain that spans approximately 300 residues and is characterized by low affinity for guanine nucleotides, yet exhibits high basal turnover. These proteins have a strong propensity for self-assembly, which is essential for their GTPase activity.^13–16^ Purified aggregates exhibit inherent GTPase activity that can be significantly enhanced (by more than 100-fold) through self-assembly on lipid membranes.^16–18^ While GTP hydrolysis is critical for membrane fission catalyzed by many dynamin-like proteins, it paradoxically triggers disassembly of the protein aggregates and the dissociation of subunits from the membrane.^3,19^ However, GTP hydrolysis cannot occur simultaneously with the assembly of the dynamin-like proteins, as this would cause premature disassembly of oligomers. GTPase activity must be highly concerted, localized, and rapid to effectively initiate the membrane fission process. While some classical eukaryotic dynamins can oligomerize around lipids in a nucleotide-free (apo) state, the mechanism for the bacterial dynamin-like protein (BDLP) from the filamentous bacterium *Nostoc punctiforme* investigated here is fundamentally different; here, GTP binding is an absolute prerequisite to unlock the compact apo-conformation and trigger the massive structural transition to the open, membrane-binding state.^20,21^

The energy derived from GTP hydrolysis promotes coordinated motions that facilitate membrane fission, ensuring that the dynamin complex remains stable long enough to catalyze the conformational changes necessary for successful membrane remodeling.^22,23^ In polymerized dynamin-like proteins, 2–4 GTP molecules can be hydrolyzed per second, while the GTP dissociation rate ranges from 10 to 100 per second.^6,24^ Assembly of a microscopically visible dynamin sheath on a membrane can take up to 1 h, whereas membrane fission can begin once 10 to 20 subunits are assembled, with final fission occurring within seconds to minutes.^25^ This highlights the dynamic nature of dynamin’s function, where initial assembly may be slow, but subsequent processes like fission occur rapidly once the necessary structural conditions are met.

The ability of dynamin-like proteins to modulate membranes requires a certain degree of structural flexibility, often coinciding with the self-assembly of the proteins to facilitate effective membrane interactions.^5,18,26^ In its basal cellular state, however, BDLP exists as a soluble, dormant monomer in the cytosol—a closed conformation that prevents premature autoassembly and maintains a functional storage pool prior to membrane recruitment. Upon activation, the protein must utilize this structural flexibility to undergo a radical conformational transition into an open state to expose its binding interfaces for membrane targeting and subsequent dimerization. Consequently, resolving the intrinsic thermodynamics of this closed-to-open monomeric transition in solution is a necessary prerequisite to understanding how the protein morphs from its resting state into its active, membrane-remodeling architecture. Thus, understanding the dynamic behavior of these proteins provides valuable insights into their mechanisms of action.

In this study, we analyze the large-scale structural transition between the open and closed states of the BDLP from *N. punctiforme*, the only dynamin-like protein for which high-quality structures are available in the Protein Data Bank (PDB) for both conformations.^20,21,27^ This BDLP contains a canonical G domain, which is separated from the neck and trunk regions by hinge 2, while hinge 1 is located between the neck and trunk and serves as the main site for the closed-open transition. These and other structural elements of BDLP are indicated in the secondary structure plot in Figure 1a. The closed and open structures are available in PDB entries 2J69 ^20^ and 2W6D,^21^ respectively, and are illustrated in Figure 1b,c. The closed structure, bound to GDP, was resolved using X-ray diffraction at a resolution of 3 Å, exhibiting an acute triangular arrangement of the G domain, neck, and trunk.^20^ Figure 1b highlights that the initial closed state is stabilized by a network of salt bridges between the G domain and the trunk, while other electrostatic interactions—particularly in the hinge 1 region—become prominent later during the closed-to-open transition. A comprehensive, residue-resolved characterization of the interactions stabilizing this closed conformation is provided in Figure S1. The open BDLP state has been resolved to 9 Å by cryogenic electron microscopy, revealing a dimer loaded with a GTP analogue.^21^ This suggests that GTP binding induces a large-scale conformational change from the closed to the open state, which doubles the length of the protein from about 10 to 20 nm. BDLP dimerization is primarily facilitated by interactions between the two G domains and some contact points between the trunk regions, while the paddle domains insert into a lipid membrane,^21^ as shown in Figure 1d. It is important to note that the GDP-bound state has a lower affinity for membranes.^27^ *In vivo*, BDLP localizes to the outer leaflet of the bacterial inner plasma membrane, where it disrupts or severely compresses this outer leaflet.^21^

**Figure 1:**
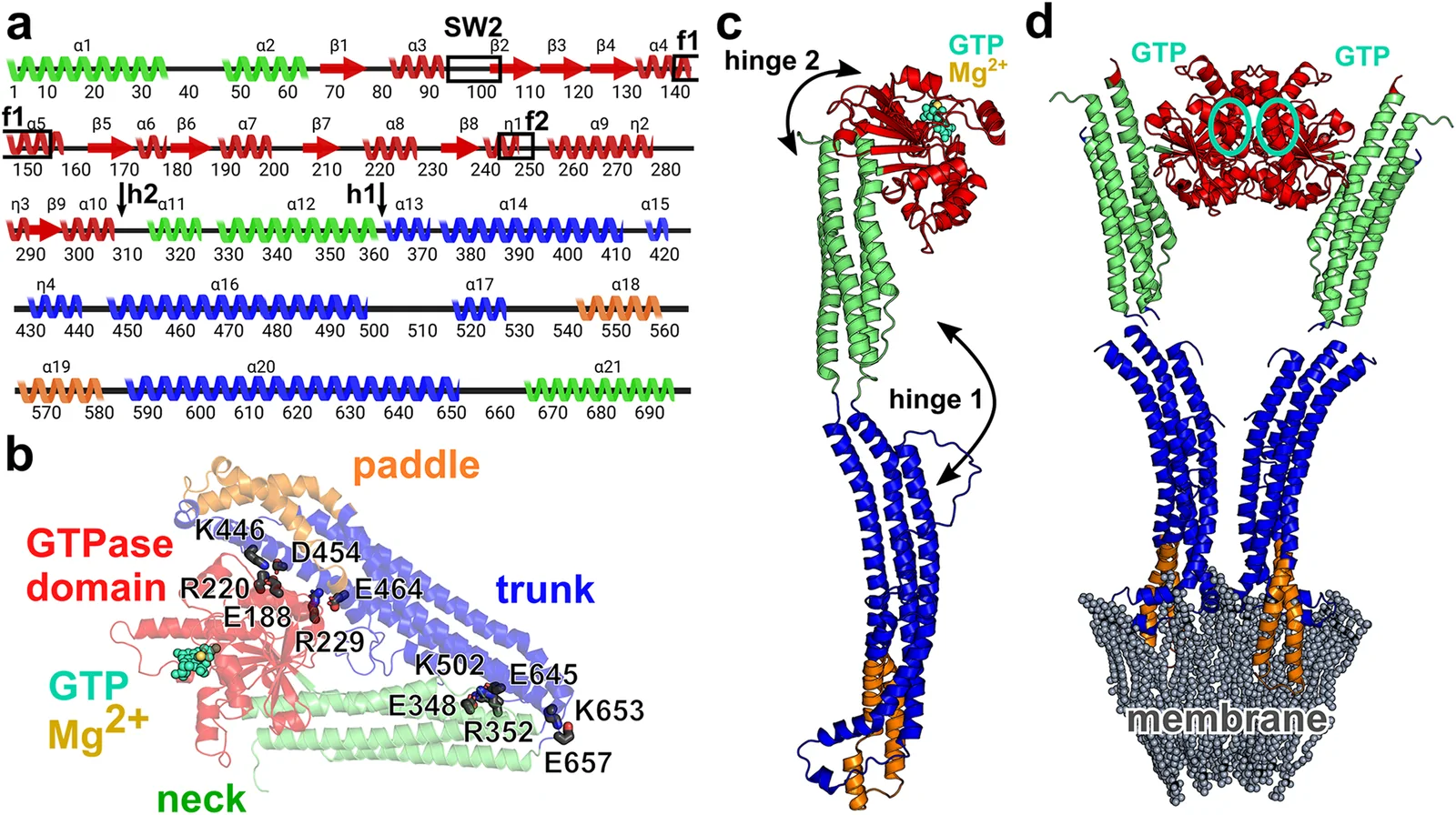
Structural characteristics of BDLP. (a) The secondary structure of BDLP is shown, including the labeling of every tenth residue number, its helices, and β-strands. BDLP contains two hinges: hinge 1 (h1) between the neck (green) and trunk (blue), and hinge 2 (h2) located between the GTPase (G) domain (red) and the neck region. In the G domain, three additional structural motifs are indicated: switch 2 (SW2), flap 1 (f1), and flap 2 (f2). (b) The closed conformation of BDLP (PDB ID: 2J69 ^20^) is shown in a domain-colored cartoon representation. Key salt bridges involved in stabilizing the closed state or guiding the subsequent opening mechanism are highlighted as sticks with explicit residue labels. A detailed sequence conservation analysis and quantitative characterization of the initial domain interface are provided in Figure S1. (c) The open form of BDLP is displayed as a monomer, highlighting the holo-state defining GTP (cyan) and Mg^2+^ (yellow) bound to the G domain. The hinge 1 and hinge 2 motions are indicated by black arrows. (d) The structure of the open form was determined for a BDLP dimer bound to a lipid membrane (PDB ID: 2W6D^21^), with the membrane colored gray. The coloring for the BDLP domains is consistent: red for the G domain (with the GTP binding site indicated by cyan ovals), green for the neck, blue for the trunk, and orange for the paddle.

While *N. punctiforme* provides the primary high-resolution structural template for BDLP, evolutionary conservation analysis reveals a stark architectural divergence across species. As a prominent member of the dynamin superfamily, BDLP exhibits high sequence and structural conservation strictly within its catalytic G domain, aligning with broader superfamily trends.^17,28^ In contrast, the surrounding neck, trunk, and paddle domains display significantly lower conservation (Figure S1). This localized variation directly impacts the domain–domain interfaces, resulting in poorly conserved interdomain contacts. Consequently, how these shifting interface properties dictate the conformational dynamics and thermodynamic transitions of the mechanochemical cycle across different species remains to be generally elucidated, an issue we address for *N. punctiforme* BDLP in the current work.

Here, we unravel the molecular details of the closed-to-open transition of BDLP and provide a rationale for the structural prerequisites and energetic implications of this radical conformational change. The primary focus of this work is the comprehensive characterization of how nucleotide binding modulates the conformational landscape of the BDLP monomer. To achieve this, we perform all-atom molecular dynamics (MD) simulations of both apo- and holo-BDLP, the latter with GTP and Mg^2+^ bound to the G domain (Figure 1c). In our previous work on guanylate-binding proteins, which belong to the dynamin superfamily, we successfully utilized MD simulations to elucidate conformational transitions, dimerization, and membrane interactions.^29–32^ To thoroughly investigate the closed-to-open pathway, we employ standard MD simulations alongside advanced enhanced sampling techniques, namely Hamiltonian replica exchange (HREMD) and umbrella sampling (USMD). This multilayered approach allows us to map the explicit free energy profiles, quantify the thermodynamic acceleration of the transition, and dissect the underlying allosteric communication network. Crucially, because the free energy profiles reveal that the open monomer remains thermodynamically unfavored, we systematically expand our scope using coarse-grained (CG) simulations to demonstrate how the active state gains necessary stability through the synergistic effects of dimerization and membrane binding. This study represents the first all-atom MD investigation of a full-length dynamin-like protein, expanding upon previous work that relied exclusively on coarse-grained architectures.^33–38^ Furthermore, this study is the first to map the complete closed-to-open transition pathway of BDLP, and dynamins in general.

## 2 Results

### 2.1 Open BDLP is Highly Dynamic and Can Adopt Semiclosed and Wide-Open Conformations

Using the BDLP from *N. punctiforme* as a portal to understanding dynamin function, we started the study by exploring the overall stabilities and local flexibilities of apo- and holo-BDLP in the open and closed states in unbiased MD simulations. The analysis of protein flexibilities, as measured by the root-mean-square fluctuations of the C_α_ atoms (RMSF), revealed that, on the simulated time scale of 600 ns, both apo- and holo-BDLP are stable in the closed conformation (Figure 2a). Nonetheless, in the closed holo-BDLP state, GTP binding induced flexibilities, which are near the GTP binding site and at the interface between the G domain and the paddle. These instabilities are caused by GTP binding, particularly involving helices α5 (residues 143–156) and α9, along with the preceding loop (residues D251–L271; see Figure 1a for the sequence). We denote these labile regions as flap 1 and flap 2 due to their swinging motions (discussed in more detail below). Interestingly, evolutionary analysis reveals that the G domain–trunk interface exhibits only moderate sequence conservation on the trunk side, remaining noticeably less invariant than the highly conserved catalytic core (Figure S1). This moderate conservation—coupled with relatively weak binding affinity at this interface, as revealed by a residue-level binding free energy analysis (Figure S1)—imparts an intrinsic thermodynamic plasticity to the region. While holo-BDLP remained closed during the MD simulation, this increased flexibility compared to the closed apo-BDLP suggests that GTP loading has the potential to drive the protein toward the open conformation.

**Figure 2:**
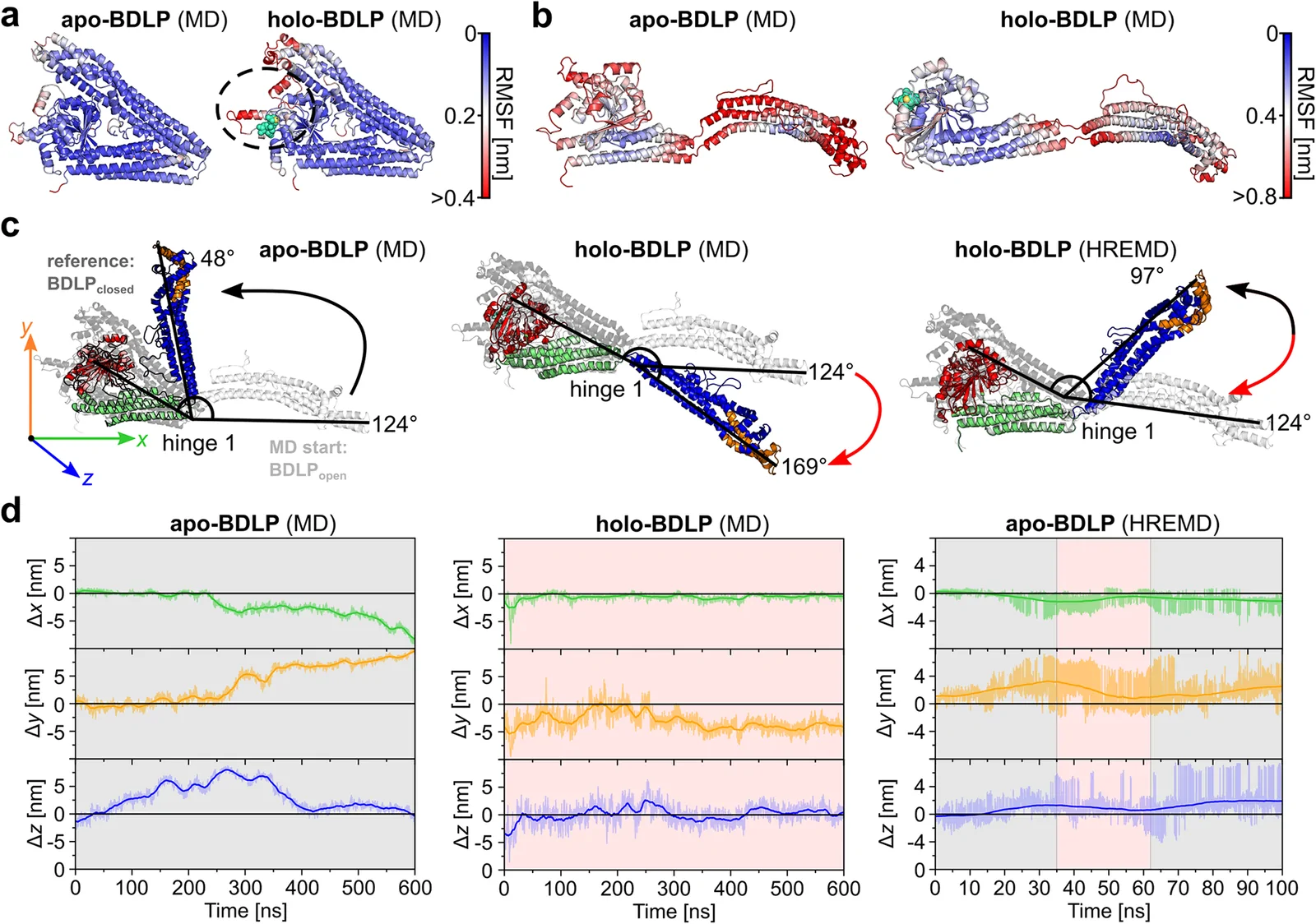
Flexibility and hinge 1 motion of monomeric BDLP. (a) The RMSF measured during 600 ns MD simulations of closed BDLP is shown for both the apo- (left) and holo-protein (right), projected onto the structures. The color scale on the right ranges from blue for low RMSF (close to zero) to red for high RMSF values of about 0.4 nm and above. The largest flexibilities in holo-BDLP were observed in structural elements (flap 1 and flap 2) near the GTP binding site and at the contact point between the G domain and the paddle, indicated by a dashed black circle. (b) The RMSF during 600 ns MD simulations of open BDLP is shown for both the apo- (left) and holo-protein (right), projected onto the structures. The color scale on the right ranges from blue for low RMSF (close to zero) to red for high RMSF values of about 0.8 nm and above. (Replicate runs shown in Figure S2). (c) The hinge 1 motion of BDLP is depicted by limiting structures and measured as the angle between residues G124, Q359, and D587. All simulations started from the open state (light gray) with a hinge 1 angle of 124°. In the 3 × 600 ns MD runs of apo-BDLP, a semiclosed conformation with 48° was reached (left), while in the 3 × 600 ns MD runs of holo-BDLP, a wide-open conformation with 169° was obtained (middle). In a 100 ns HREMD simulation of holo-BDLP, the protein oscillated between semiclosed conformations (up to 97°) and the open state (right). Closing motions are indicated by black arrows, and opening motions by red arrows. The fully closed state is shown as a reference in dark gray cartoon. (d) The hinge 1 motion is further characterized by movements of the stalk tip, residue G580, in Cartesian space. The coordinate system definition is given in (c), and the changes Δ*x* (green), Δ*y* (orange), and Δ*z* (blue) relative to the starting position of G580 in the open state are plotted for the 600 ns MD of apo-BDLP (left), holo-BDLP (middle), and for the 100 ns HREMD of holo-BDLP (right). Phases of closing and opening for the direction of hinge 1 are indicated by gray and pink backgrounds, respectively. (Replicate runs shown in Figure S5).

The RMSF values obtained from MD simulations of the open BDLP state show that GTP binding stabilizes the open form, as the RMSF values for holo-BDLP are smaller than those for apo-BDLP (Figure 2b). For the apo-BDLP, the largest RMSF values are observed in the trunk and paddle, and the G domain is also more mobile in the open apo-BDLP, indicating that the open conformation and the apo-state are not compatible with each other. However, it should be noted that the open state is generally more mobile than the closed state, as the RMSF values for both apo- and holo-BDLP in the open state are twice as large compared to the closed forms. Replicate MD simulations for each system yielded consistent results (Figure S2).

Visual inspection of the MD trajectories revealed that, in the absence of GTP, the open state of BDLP begins to convert toward the closed state, reaching a semiclosed conformation after just 200 ns of simulation time. To quantify this closing motion around hinge 1, we measured the angle between the C_α_ atoms of residues G124, Q359, and D587 across all three independent MD runs per system (Figure 2c, left). At the beginning of the simulation, initiated from the open conformation, this hinge 1 angle is approximately 124°, decreasing to 48° in the semiclosed state. In contrast, the open holo-BDLP remained open during the 600 ns simulation, with the trunk moving in the opposite direction and assuming hinge 1 angles of up to 169°(Figure 2c, middle). We refer to this conformation as the “wide-open conformation”. In contrast, the accompanying movements at the opposite end of the protein—specifically the G domain relative to the neck—are far less pronounced, with close-up views of this hinge 2 motion provided in Figure S3 for completeness.

To further quantify the hinge 1 motion, we measured the displacement of the stalk tip, with the stalk defined as the trunk plus paddle, at residue G580 relative to its initial position in the open state. The spatial distribution of this residue across each simulation is shown in Figure S4. Changes in *x* and *y* of G580, using the definition of the Cartesian coordinate system shown in Figure 2c, reflect the hinge 1 motion, where negative Δ*x* and positive Δ*y* indicate the closing direction, and the opposite for the opening direction. The closing motion is observed in the MD simulation that started from the open apo-BDLP, while further opening is indicated by the Δ*x* and Δ*y* values of holo-BDLP (Figure 2d, left and middle). Importantly, replicate MD simulations captured similar G580 displacement profiles, confirming the reproducibility of these distinct hinge 1 motions (Figure S5).

To achieve enhanced sampling of the hinge 1 conformational space, we performed HREMD simulations with 100 ns per replica of apo-BDLP, starting from the open state. As in the MD simulation, no complete closing was observed. Instead, the HREMD simulation also sampled a semiclosed state, reaching a hinge 1 angle of only 97° (Figure 2c, right). However, the Δ*x* and Δ*y* values in Figure 2d (right) indicate that the closing motion was reversed at ≈35 ns, resulting in the opening of BDLP again, before another closing phase started at ≈60 ns.

We conclude that BDLP is a flexible protein. While closed apo-BDLP and open holo-BDLP are both considerably stable—particularly the former—closed holo-BDLP harbors instabilities that may drive it to open, whereas open apo-BDLP tends toward the closed conformation. Importantly, this global flexibility does not stem from structural denaturation; DSSP analysis^39^ of the independent MD replicates confirms that the core helical framework of the neck and trunk remains remarkably stable throughout the trajectories (Figure S6). Minor conformational fluctuations are confined strictly to the loops at the tips of these helix bundles, leaving the overall domain geometries intact.

### 2.2 Hinge 1 Is Not Just a Hinge But a Restricted Ball Joint

For Figure 2d, we calculated not only the changes in *x* and *y* for the motion of the stalk tip but also whether the stalk tip moves in the lateral direction represented by *z*. For a pure hinge motion, we would not expect changes along this direction. However, the closing motions observed in the MD and HREMD simulations of open apo-BDLP were accompanied by lateral motions of the stalk. In the MD simulation, Δ*z* values exceeded 5 nm (Figure 2d, left). Interestingly, motions along *z* preceded the closing motion of the stalk, as Δ*z* began to rise at ≈50 ns, while Δ*x* and Δ*y* did not show significant changes until 250 ns onward. At that point, the motion along *z* reversed, and at about 400 ns, the stalk tip returned to its starting position in the *z* direction (i.e., Δ*z* ≈ 0), with no further noteworthy lateral motions occurring for the remainder of this MD simulation. These observations imply that hinge 1 is not simply a hinge, as its name suggests, but resembles a restricted ball joint.

A broader and more quantitative overview of the conformational landscape of BDLP was obtained through the calculation of free energy surfaces from the 3 × 600 ns MD simulations of the open forms of apo- and holo-BDLP. In this analysis, the free energy (Δ*G*) is projected onto various combinations of order parameters, according to eq 1 in the Methods section. Along with the previously introduced hinge 1 angle and Δ*z*, we defined an angle for hinge 2, formed by the residues S291, P303, and L323 (Figure 3a). Additionally, we measured the distance between residues N224 and T453, referred to as *d*_GT_, to assess the extent of opening and closing between the G domain and trunk.

**Figure 3:**
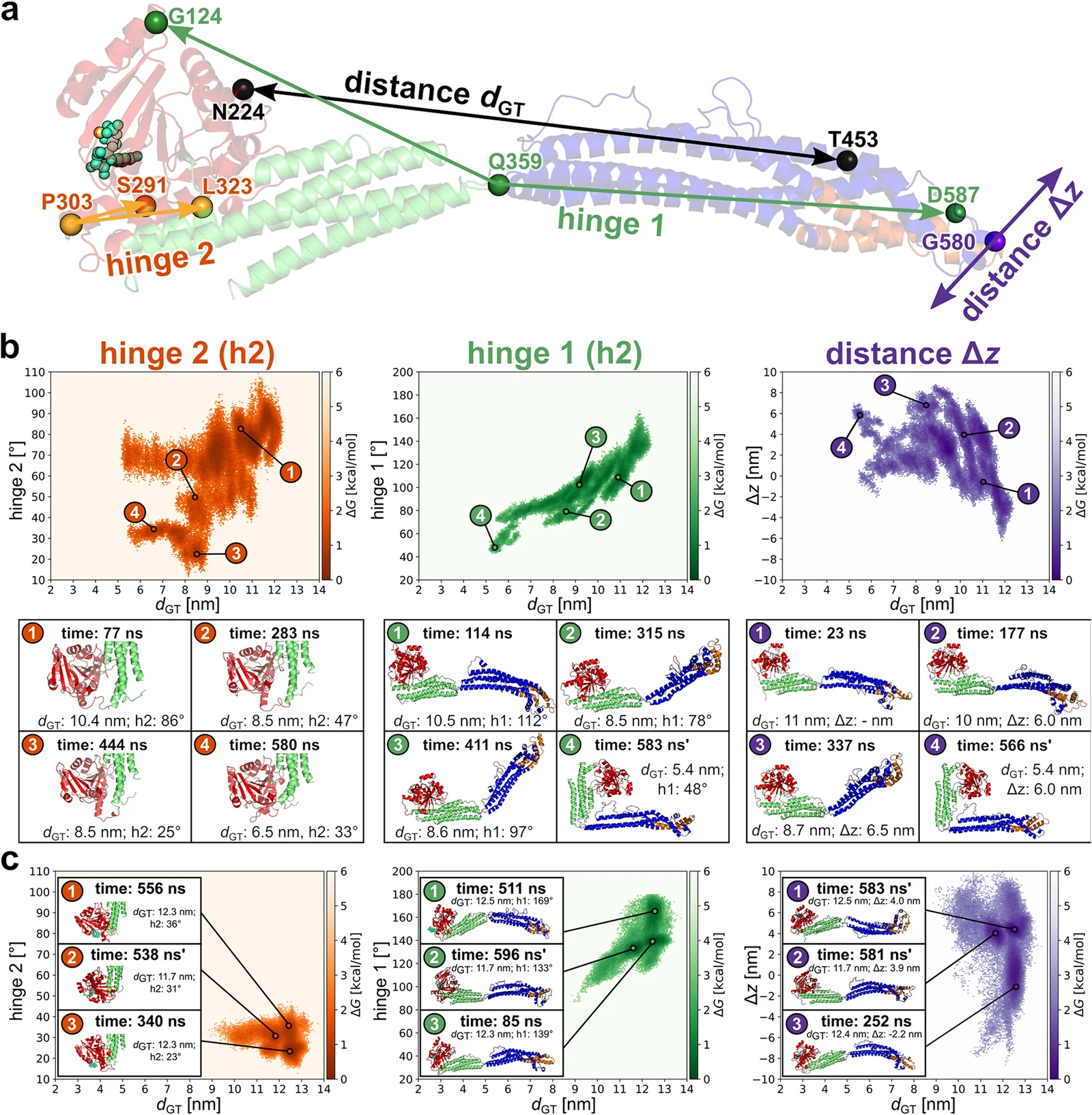
Free energy surfaces to characterize the hinges and lateral trunk motions in the open BDLP. (a) Definition of the order parameters used for projecting the free energy Δ*G* (from left to right): hinge 2 (h2) between the G domain and the neck, hinge 1 (h1) between the neck and the trunk, the distance *d*_GT_ between the G domain and the trunk, and the lateral motions Δ*z*. The free energy is shown as 2D projections Δ*G*(*d*_GT_, h2) (left), Δ*G*(*d*_GT_, h1) (middle), and Δ*G*(*d*_GT_,Δ*z*) (right), along with representative structures for (b) apo-BDLP and (c) holo-BDLP. Δ*G* values in kcal/mol are color-coded according to the scales on the right of the plots. Low Δ*G* values indicate stable structures. The times at which the representative structures were sampled during the triplicate 600 ns MD simulations of apo- and holo-BDLP, both initiated from the open state, are provided. Primes indicate conformations sampled from one of the replicate runs.

We start with the analysis of the free energy surfaces for apo-BDLP shown in Figure 3b, complemented by representative structural snapshots of the populated states displayed below the surfaces. The Δ*G*(*d*_GT_, hinge 1) values (middle panel) confirm that it closes spontaneously to reach the semiclosed state, resulting in reductions in both *d*_GT_ and hinge 1 values. However, for the same angle, different distances between the G domain and trunk are possible. For instance, when hinge 1 has an angle of about 80°, the distance *d*_GT_ can be around 6 nm or between 8 and 9 nm. This degeneracy indicates that motions in another direction must occur in parallel, which are captured by Δ*z* representing the lateral dimension. The projection Δ*G*(*d*_GT_, Δ*z*) (right panel) shows that as apo-BDLP closes, the trunk initially moves in the positive Δ*z* direction; then, at *d*_GT_ ≈ 9 nm, it starts to move in the opposite direction. This Δ*z* motion supports the conclusion that hinge 1 operates like a ball joint. The comparison of the three free energy projections in Figure 3b reveals that the two hinge motions and the lateral movement of the trunk are interconnected, as changes in these dimensions occur simultaneously.

Next, we examine the effects of GTP binding on the free energy surfaces, which are presented for holo-BDLP in Figure 3c. This quantitative analysis reinforces our earlier observation that GTP binding stabilizes the open conformation and promotes a transition to a wide-open conformation. The distance between the G domain and trunk consistently measures between 12 and 13 nm in holo-GTP, regardless of whether the state is open or wide-open, while hinge 1 increases from 130° to 180°. Such high values for both *d*_GT_ and hinge 1 were not observed in apo-BDLP. Additionally, similar to apo-BDLP, lateral motions of the trunk also occur in holo-GTP, as shown by the projection Δ*G*(*d*_GT_, Δ*z*) (right panel). Although no closing of holo-BDLP occurs, changes in hinge 2 are still evident, suggesting that the transition from the open to wide-open conformation induces structural changes at the opposite end of the protein. However, these changes in hinge 2 are minimal, as illustrated by the zoomed images of that protein region in Figure S3, with hinge 2 values shifting only from approximately 40° to 20°, as shown in the Δ*G*(*d*_GT_, hinge 2) projection in Figure 3c (left panel).

### 2.3 GTP Binding Ignites Communication across BDLP’s Structure

It is understood that GTP binding to the closed state of BDLP prompts the protein to transition to an open conformation. To explore the origin of the coupling between these events, we analyzed the structural changes in the G domain upon GTP binding and how these alterations are transmitted across the protein.

The RMSF analysis presented in Figure 2a,b demonstrates that, upon GTP binding, the G domain becomes more flexible in the closed conformation and more rigid in the open conformation. To quantify this, we applied conformational clustering to the G domain motifs: hinge 2, flap 1, flap 2, and switch 2. For the open BDLP conformation, we identified a total of 103 clusters for these motifs in holo-BDLP, whereas this number increased 6-fold for apo-BDLP, resulting in 624 clusters with considerable variance among the conformations of hinge 2 and flap 2. In contrast, for the closed form of BDLP, the GTP-bound state displayed 178 clusters with high conformational variance in hinge 2 and flap 2, while only 61 clusters were observed for apo-BDLP. These results suggest that the open conformation can more effectively accommodate GTP, whereas in the closed conformation GTP induces structural instabilities. To characterize the motions of these motifs, principal component analysis was applied to their trajectories in the MD simulations of closed and open BDLP, both with and without GTP bound. This analysis revealed swinging motions for flap 1 and flap 2 (Figure 4a), motivating our naming for them. In particular the closed conformation of holo-BDLP displays pronounced flap 1 and flap 2 movements to cover GTP.

**Figure 4:**
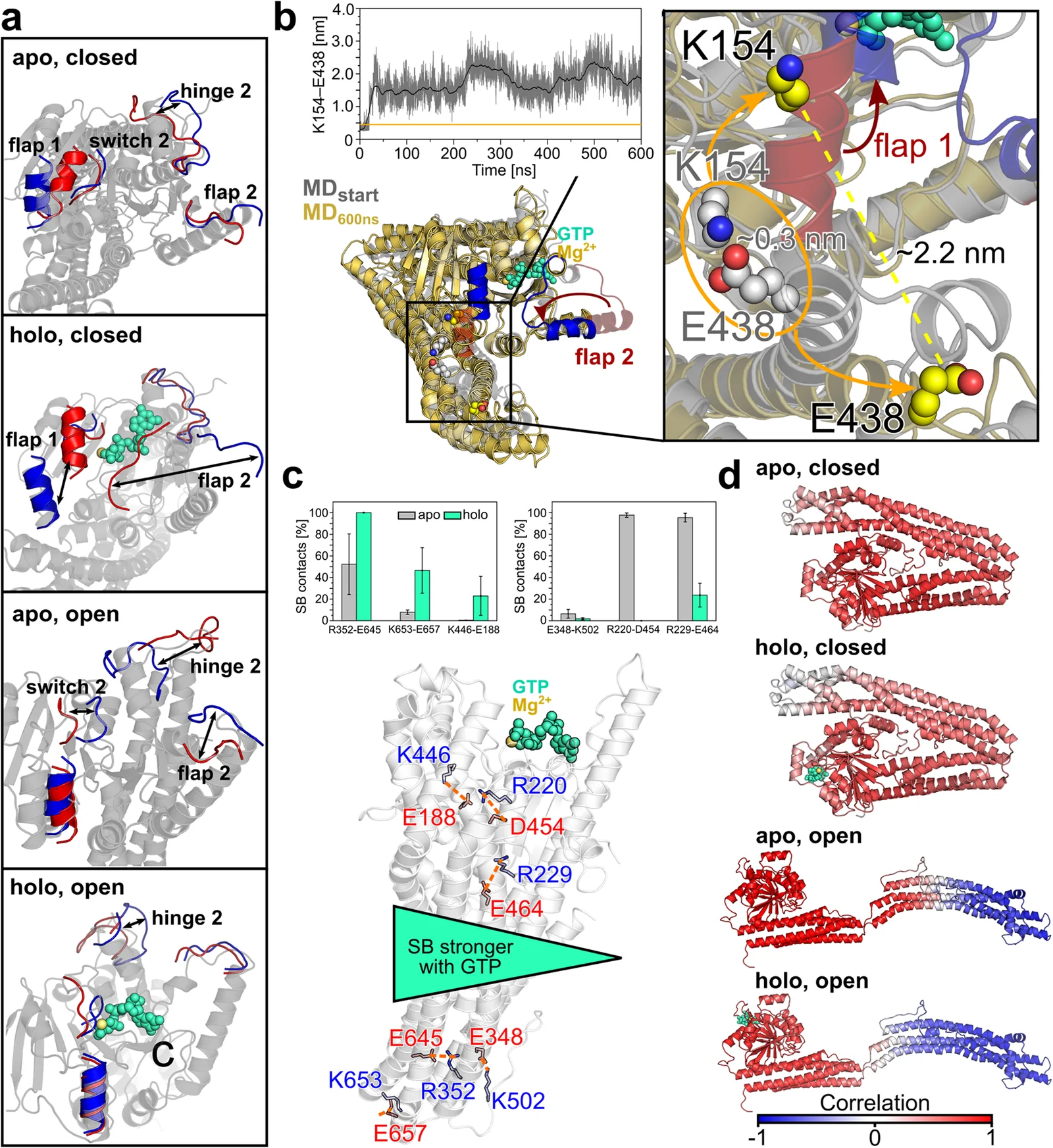
Conformational and salt bridge changes caused by GTP binding. (a) Principal motions of key structural elements in the G domain of closed and open apo- and holo-BDLP. Maximum displacements for flap 1, flap 2, hinge 2, and switch 2 are shown in blue and red, with large motions indicated by arrows. (b) Breaking of the salt bridge (SB) K154–E438 upon GTP binding to closed BDLP. (Top) The evolution of the distance between the N atom of the Lys side chain and either O atom of the Glu side chain over 600 ns MD; the SB is considered formed when this distance is below 0.45 nm (orange line). (Bottom) Comparison of the MD starting structure (gray cartoon) with the structure at 600 ns (yellow cartoon) shows the breaking of the salt bridge (within the black square), caused by the movement of flap 1 from its initial (red) to final (blue) position in response to GTP binding. (Right) Zoom into the K154–E438 region shows the distance increasing from approximately 0.3 to 2.2 nm by the end of the simulation. (c) Occupancies of salt bridges that strengthen (left) or weaken (right) as a result of GTP binding (top), located on different longitudinal sides of the trunk (bottom), leading to a shearing motion. Results are averaged over the triplicate 600 ns MD runs (standard errors shown as bars) of closed apo- and holo-BDLP, shown in gray and green, respectively. (d) Correlation of motions within BDLP is color-coded for different protein states, ranging from blue for anticorrelation (−1) to red for correlation (+1). Results are derived from 600 ns MD of each state.

After analyzing the structural changes within the G domain following GTP binding, we further investigated how these changes are communicated to the other domains, particularly the neck and trunk. To accomplish this, we identified salt bridges that alter their occupancies upon GTP binding in the MD simulations of closed BDLP. The most obvious candidate that might function as an allosteric switch is a salt bridge connecting flap 1 with the trunk, K154–E438, which is dissolved by a flap 1 relocation following GTP binding. This causes flap 1 and thus K154, which is part of this flap, to move toward the GTP and thus away from E438, which is located next to the paddle domain (Figure 4b). There was breakage of two further salt bridges between the G domain and the trunk at R220–D454 and R229–E464 (see Figure 1b for the locations of these residue pairs within the 3D structure). Of note also, GTP binding also reduced the stability of the neck-to-trunk salt bridge E348–K502. Interestingly, these residue pairs are all on the same side of BDLP (which is the right side in the protein presentation in Figure 4c). Contrariwise, on the other side of the protein, three salt bridges gained in strength upon GTP binding: E188–K446 connecting the G domain with the trunk, R352–E645 between the neck and the trunk, and K653–E657 associating the loop region between the trunk and neck with the paddle domain.

This dichotomous weakening or strengthening of salt bridges across the protein demonstrates that GTP binding leads to conformational changes in the closed BDLP, likely facilitating the transition from closed to open. The salt bridges thus function as important allosteric switches that enable the transfer of information from the GTP-binding site in the G domain to various parts of the neck and trunk regions. Furthermore, the two-sided distribution of weakened or dissolved versus strengthened or newly formed salt bridges along the neck and trunk explains the lateral (or shear) movements of the trunk that accompany its opening or closing motion.

The coupling between the different protein parts is further highlighted by a dynamic cross-correlation analysis of the MD trajectories. Such analysis provides insights into how specific regions of a molecule, here BDLP, move in relation to one another. Positive correlation indicates that the movements are in the same direction, while negative correlation suggests that they move in opposite directions. In Figure 4d, these (anti)­correlations are mapped as colors on the closed and open BDLP conformations, with further distinctions made between results obtained without and with GTP bound. More detailed versions of the same results are presented as 2D plots in Figure S7. For both the closed and open conformations, GTP binding influences the correlations between the movements of different domains. In the open state, the existing anticorrelation between the motions of portions of the trunk and paddle relative to the G domain and neck becomes more pronounced upon GTP binding, extending to encompass the entire trunk. This opposing motion of the trunk relative to the neck results in the wide-open conformation. In the closed form, the GTP-induced changes are more subtle, yet they are crucial for the closed-to-open transition. The disruption of salt bridges between the G domain and the trunk leads to the paddle and tip of the trunk to start moving away from the G domain, which can be seen from their motions becoming anticorrelated relative to the G domain in closed holo-BDLP. Thus, it is the alteration of salt bridge interactions that facilitates opening of holo-BDLP, highlighting their role in allosteric communication and regulating the conformational transitions.

### 2.4 GTP Binding Halves the Energy Needed for the Closed-To-Open Transition

Since the standard MD and HREMD simulations were unable to adequately sample the entire closed-open pathway and only reached the semiclosed state, we employed umbrella sampling (USMD) to model this transition in both apo- and holo-BDLP and calculate the corresponding free energy, also known as the potential of mean force.^40^ To generate initial structures for the USMD windows, we conducted a pulling simulation of apo-BDLP along the collective variable *d*_GT_, which monitors the distance between the G domain and the trunk (Figure 3a), to enforce the closed-to-open transition. Figure S8a shows the applied pulling force. For initializing the actual simulations for each USMD window, we utilized structures previously sampled in standard equilibrium MD simulations whenever available, and supplemented any gaps in the unsampled closed-open regions with structures obtained from the pulling simulations. The reasoning for this choice is that standard MD simulations yield more equilibrated conformations, providing better starting points for USMD compared to the conformations obtained from the pulling simulations. We simulated about 70 intermediate conformations between *d*_GT_ ≈ 0.8 nm, which is the neck–trunk distance of the closed conformation, and *d*_GT_ ≈ 11 nm for the open state. These windows were restrained with harmonic potentials, as illustrated in Figure S8b, which shows the distribution of the windows. They were then simulated for 100 ns.

The potential of mean force, obtained from applying the weighted histogram analysis method (WHAM) to the USMD simulations data of apo-BDLP, is shown in Figure 5 (for the error estimate, see Figure S8c). The complete opening of apo-BDLP requires more than 14.5 kcal/mol as this was the maximum of the free-energy profile. The initial steep rise in the energy profile between *d*_GT_ = 1 and 4.5 nm can be attributed to the breaking of several salt bridges between the neck and trunk. Per-residue binding free energy calculations show that these neck–trunk interactions are stronger than those between the paddle and G domain, explaining why the energy profile continuously increases throughout the transition rather than just at the very beginning. At *d*_GT_ ≈ 5.3 nm, there was a metastable conformation corresponding to the semiclosed state already sampled in the MD and HREMD simulations. This structure is stabilized by a trunk loop that involves a salt bridge between D490 and E510, along with a β-hairpin that makes contact with the G domain. Once this contact is broken, the opening proceeds until an energy valley corresponding to an almost fully opened apo-BLDP conformation with *d*_GT_ ≈ 9.4 nm is achieved.

**Figure 5:**
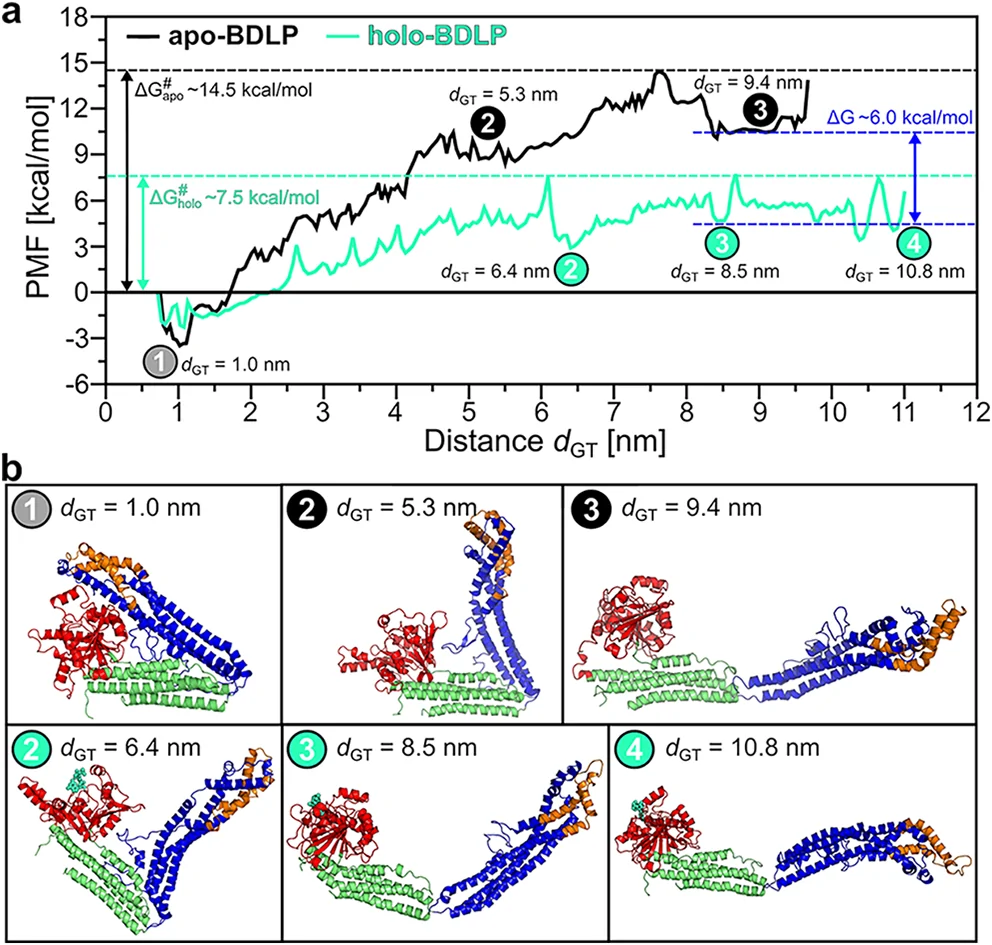
The potential of mean force (PMF) of the closed-to-open transition of BDLP. (a) The PMF profile was obtained from USMD simulations for apo-BDLP (black) and holo-BDLP (cyan), using the distance *d*_GT_ as the collective variable. The corresponding free energy barriers Δ*G* ^#^ for the closed-to-open transitions (left) and the difference in Δ*G* for the open states of apo- and holo-BDLP (in blue on the right) are indicated. (b) Representative structures of local minima along the opening pathway, obtained via clustering analysis of the corresponding windows at the specified *d*_GT_ values, are shown and their positions are marked in (a). Structure 1 represents both apo- and holo-BDLP.

Next, we repeated the USMD simulations after docking GTP to the binding site of the initial conformation of each window and adding Mg^2+^. The potential of mean force after 100 ns per US window is also shown in Figure 5a (for the error estimate, see Figure S8c). The overall energetic threshold for the closed-to-open transition is lowered by about 7 to 7.5 kcal/mol. Thus, the binding of GTP almost halves the energy needed for opening up the protein structure and also renders the open conformation more favorable, as the difference in free energy for the open conformations is 6.0 kcal/mol. As observed above, the addition of GTP sets off several structural changes in the G domain, which in turn break key salt bridges between the G domain and the trunk, enabling the opening at hinge 1. However, hinge 2 is also affected by GTP binding, which causes the G domain to roll away from the neck. This movement lowers the energy barrier for dissolving the interaction between the trunk loop and the G domain that is still present in the intermediate at *d*_GT_ ≈ 6.4 nm. Once these contacts have been broken, not much energy is required to reach the semiclosed state at *d*_GT_ ≈ 8.5 nm, which was previously identified as a metastable state in the MD simulations, yet for apo-BDLP (see Figure 5b). From there, further opening of holo-BDLP takes place until fully open states with *d*_GT_ ≈ 10.8 nm are reached.

The energy required for the opening of apo-BDLP and holo-BDLP is about 14.5 and 7.5 kcal/mol, respectively, which in principal could be supplied by the hydrolysis of a single GTP molecule.^41,42^ However, GTP hydrolysis should only occur after self-assembly and membrane binding of BDLP for membrane remodeling to become possible. Therefore, in addition to focusing on energy levels and free energy accessibility within molecular processes, it is equally important to consider the time scales involved. Even if a process is energetically accessible, it may not occur if the kinetics are too slow, allowing alternative reactions to dominate in the meantime. The application of the Arrhenius equation (eq 2 in the Methods section) enables us to convert the free energy barriers of 14.5 and 7.5 kcal/mol into the time scale required for the closed-to-open transition. It takes more than 30 ms in apo-BDLP, while this duration reduces to about 250–300 ns for holo-BDLP. The conformational change of BDLP can thus take place more quickly than GTP hydrolysis and it can occur before the dimer dissociates, all of which is well below the seconds to minutes reported for membrane fission itself.^25^ A relevant question is why we did not sample the transition in the 600 ns MD or HREMD simulations of holo-BDLP. The likely explanation is that USMD focuses on a targeted region of the energy landscape between two end states, while unbiased simulations allow the protein to explore a broader conformational space, which may not lead directly to the expected end state.

### 2.5 Membrane Anchoring Delays But Does Not Prevent Partial BDLP Closure

The free energy profiles in Figure 5 showed that the open states of BDLP are not as stable as the closed conformation, even after GTP binding. We therefore carried out further simulations to determine whether the open BDLP state is more stable in the membrane-bound state or as a dimer. Given the size of these systems, we applied coarse-grained MD simulation using the Martini 2.2 force field. The lipid membrane was modeled as a 1-palmitoyl-2-oleoyl-*sn*-glycero-3-phosphocholine (POPC) bilayer. We simulated seven systems: (i) closed monomer in solution, (ii) closed monomer on the membrane, (iii) closed dimer in solution (this system was considered for comparison), (iv) open monomer in solution, (v) open monomer on the membrane, (vi) open dimer in solution, and (vii) open dimer on the membrane (Figure 6). The closed structures were simulated for 1 μs and the open structures for 2 μs.

**Figure 6:**
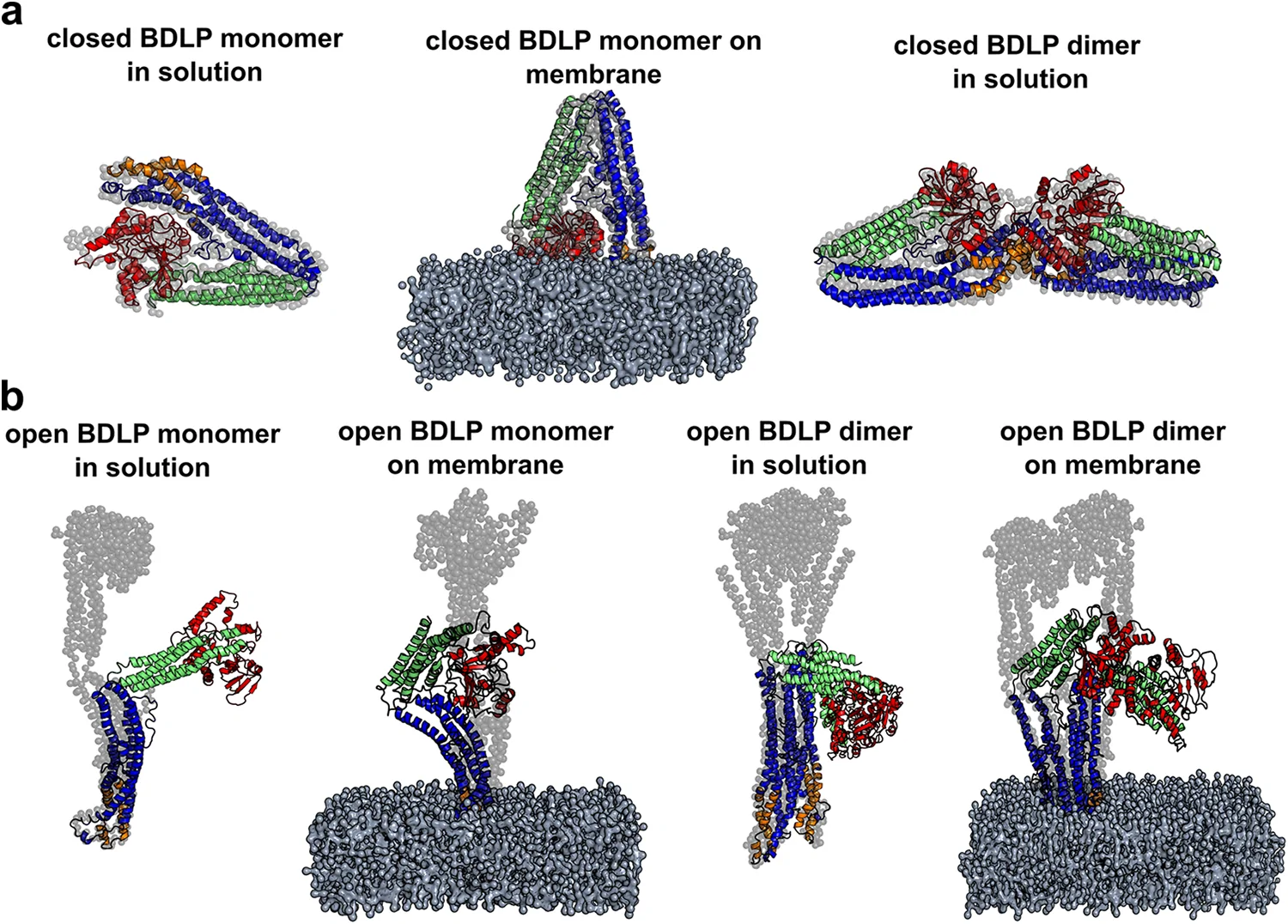
Effects of membrane binding and dimerization on the stability of BDLP. The results of coarse-grained MD simulations of (a) closed structures simulated for 1 μs and (b) open structures simulated for 2 μs are presented. The starting structures are shown as gray cartoons, and the final structures per simulation are shown in color after backmapping the last protein frame to the all-atom representation. The POPC membrane is shown as a gray van der Waals surface.

Consistently with the previous all-atom simulations (Figure 2), the closed monomer in solution proved to be highly stable at the coarse-grained level. This stability extends to the closed dimer in solution, with both systems exhibiting only minor fluctuations around their starting configurations. Similarly, the closed monomer remained stable when bound to the membrane. It also stayed in close contact with the membrane, including the G domain, as some of its helices are sufficiently hydrophobic to dip into the membrane core. We expect this G domain–membrane interaction to further stabilize the closed BDLP conformation.

When starting the simulation of the membrane-bound BDLP monomer from its open conformation, it adopts a semiclosed state within 1 μs, closely resembling the behavior of the open monomer in solution. A similar trend is observed for the BDLP dimer, both in solution and on the membrane, where both protomers assume a semiclosed structure while maintaining their dimeric interface. Interestingly, the 2 μs trajectories revealed that membrane anchoring delays this transition; while the solution dimer closes rapidly, the membrane-bound dimer exhibits more stability in the open state for most of the trajectory before displaying initial instabilities and partial closure toward the end of the 2 μs window. These observations highlight the cooperative nature of BDLP stabilization. While membrane insertion and initial dimerization are clearly crucial factors that delay closure, the late-stage instabilities observed in the dimer underscore that these two components alone are not sufficient to permanently lock the open conformation. Instead, they point to a model where higher-order BDLP polymerization is required to fully stabilize the open state on the membrane.

## 3 Discussion

This study offers new insights into the dynamics of the bacterial dynamin-like protein (BDLP) from *N. punctiforme*. By combining an analysis of the dynamics of different BDLP conformations and salt bridge interactions with our understanding of the free energies required for the transition from closed to open states, we elucidated the specific requirements for BDLP to achieve and maintain the open conformation following GTP binding.

We determined that the initial energetic barrier for BDLP opening arises primarily from disrupting salt bridges between the G domain, neck, and trunk, leading to a semiclosed intermediate. Once these electrostatic interactions are severed, less force is required to achieve full opening. This transition involves both a hinge 1 motion and a lateral shearing that minimizes opposing electrostatic forces. GTP binding lowers the opening barrier by 5 orders of magnitude through an allosteric mechanism initiated by two flaps closing over the nucleotide site. This flap closure disrupts G domain–trunk salt bridges, initiating paddle detachment, while new salt bridges form on the opposite side of the trunk to guide the stalk’s lateral motion. Concurrently, releasing neck–G domain salt bridges allows the G domain to roll away at hinge 2. By mapping out these steps, our results agree with Low et al.^21^ that GTP binding is essential for hinge 1 opening, while revealing the precise molecular mechanism behind this transition.

However, further hinge 1 opening coincides with a structural relaxation of the GTP binding pocket at switch 2. This explains why GTP hydrolysis by dynamins typically requires polymerization, where adjacent G domains stabilize one another to provide catalytic competence. Beyond clarifying hydrolysis dynamics, revealing this allosteric salt-bridge reshuffling along the protein trunk highlights a potential strategy for targeted pharmacological intervention. Small molecules or allosteric modulators could be designed to target these transient trunk pockets and stabilize the closed-state salt-bridge network. By maintaining these interactions, such molecules could trap the monomer in its autoinhibited conformation even in the presence of GTP, effectively preventing the mechanical transition required for membrane attachment and assembly.

The potential of mean force of the closed-open transition in Figure 5 further shows that the open state of the BDLP monomer in solution is less stable than the closed state. We also demonstrated that while the combined effects of BDLP dimerization and membrane binding promote open-state stability, they are not sufficient on their own to fully lock the open conformation. Our results thus clarify why Low et al.^21^ only observed the open form for holo-BDLP when being membrane-bound and polymerized. The GTP-dependent face-to-face dimerization considered in our BDLP simulations represents a universally conserved structural mechanism within the dynamin superfamily. While capturing full-length, extended dimers with nucleotide bound remains experimentally challenging for many eukaryotic and bacterial homologues due to their high conformational flexibility and tendency to polymerize, the core G-domain dimeric interface is a well-documented structural hallmark of activation. This nucleotide-driven face-to-face assembly has been structurally characterized in various organisms, including eukaryotic systems such as human Dynamin-1 ^44^ and human MxA,^45^ as well as bacterial homologues like DynA from *Bacillus subtilis* ^46^ or synDLP from *Synechocystis sp.*.^47^ Our simulations of the full-length BDLP dimer thus provide valuable atomistic insights into this widely conserved superfamily mechanism that is otherwise difficult to trap experimentally in its fully extended form.

Layering our simulation data onto established experimental milestones enables us to propose a refined model for BDLP’s mechanochemical cycle (Figure 7). The cycle begins with the cytosolic monomer. Our equilibrium and enhanced sampling simulations demonstrate that GTP binding directly weakens the autoinhibitory contacts between the paddle and the G domain, significantly lowering the activation barrier for opening. In the second step of the cycle, the closed, GTP-bound BDLP targets the lipid membrane. Importantly, our structural analysis of the membrane-bound closed state reveals that the lipid bilayer physically blocks the nucleotide-binding pocket from the solution phase. This steric hindrance leads us to conclude that GTP binding must chronologically precede membrane attachment. In the third step, membrane interactions break the remaining paddle–G domain contacts, allowing the monomer to relax into a half-open conformation on the bilayer. Our free energy profiles show that GTP binding reduces energy barrier for the opening transition. Our subsequent simulations of the membrane-bound dimer (step 4) demonstrate that membrane interactions and dimerization act together to thermodynamically strengthen this half-open architecture. Our simulations highlight that lateral motions of the trunks are structurally crucial here to prevent steric collisions during hinge 1 opening within the dimer. The subsequent stages of the cycle (steps 5 and 6) rely on well-documented experimental literature for proteins from the dynamin superfamily.^21,25^ These membrane-bound dimers assemble into larger polymers that remain stable until GTP hydrolysis occurs. After GTP hydrolysis, GDP leaves the binding site (step 6, known from ref 20) and the process is reversed. As established by FRET-based bulk vesicle assays, BDLP does not catalyze membrane fusion but rather deforms, bends, and tubulates the bilayer.^43^ When the closed BDLP finally dissociates from the membrane, the thinned, highly curved membrane structure is destabilized, facilitating downstream remodeling.

**Figure 7:**
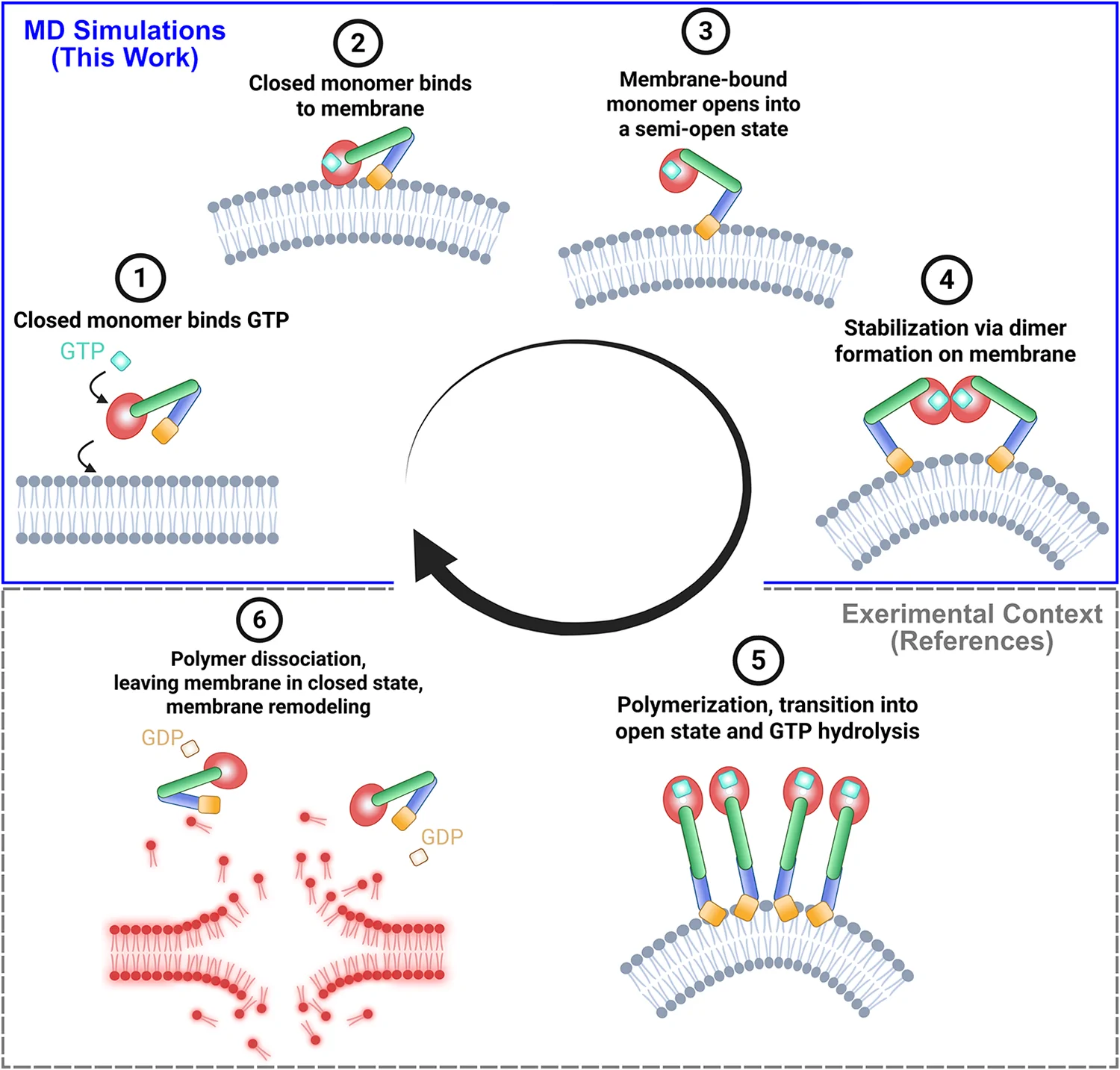
Proposed model for the mechanochemical cycle of BDLP. Schematic representation integrating our computational findings (enclosed in the blue box) with established experimental literature (enclosed in the gray-dashed box) to illustrate the interplay between nucleotide states, membrane binding, and conformational changes. Steps 1–4 are directly supported by our MD simulations and free energy profiles, demonstrating that (i) GTP binding must precede membrane attachment due to steric blocking of the nucleotide pocket in the bound closed state, (ii) GTP binding destabilizes the autoinhibited monomer to permit initial membrane binding, and (iii) membrane interactions combined with (iv) dimerization act together to stabilize the half-open conformation. Steps 5–6 show downstream stages (polymerization, GTP hydrolysis, and membrane deformation/release), which are derived from established experimental frameworks.^20,21,25,43^ Figure created with BioRender.com.

## 4 Conclusion

By combining multiscale standard and enhanced sampling molecular dynamics simulations totaling over 37 μs, this study establishes the atomistic principles governing the activation and conformational dynamics of BDLP. We demonstrate that the energetic barrier for BDLP opening is dictated by a specific network of salt bridges between the G domain, neck, and trunk, which are disrupted via a combined hinge 1 motion and lateral shearing. GTP binding acts as a potent kinetic accelerator, lowering the opening energy barrier by 5 orders of magnitude through an allosteric flap-closure mechanism that reorganizes these salt bridge contacts along the protein neck. Furthermore, our structural analyses reveal that membrane docking sterically blocks the nucleotide-binding pocket in the closed state, dictating that GTP binding must chronologically precede membrane insertion. Finally, our free energy landscapes demonstrate that neither membrane binding nor face-to-face dimerization alone fully locks the extended open state, but rather act synergistically to stabilize the half-open intermediate prior to higher-order assembly. Together, these quantitative thermodynamic insights clarify the fundamental mechanochemical triggers of the dynamin superfamily.

## 5 Methods

### 5.1 Overview of the Simulations

For all MD simulations, we used GROMACS.^48^ For the all-atom simulations, the Amber ff99SB*-ILDNP^49^ and the TIP3P water model^50^ were applied, while Martini 2.2 ^51^ was used for the coarse-grained simulations (CGMD). The temperature in these simulations was set to 303 K (30 °C). The HREMD simulations^52^ were conducted at 310 K and were carried out with PLUMED^53^ in conjunction with GROMACS, using 30 replicas with λ values between 1 and 0.667. The USMD simulations (at 303 K) were performed with GROMACS, running 74 × 100 ns windows for apo-BDLP and 67 × 100 ns windows for holo-BDLP. In Table 1, all simulations performed in this study are summarized, including their simulation type, starting conformation, system size, and simulation length. All fine details of the simulation settings are provided in the Supporting Information. The accumulated simulation time is 37.1 μs.

**Table 1:** Simulations Performed in This Study

Simulation type	Conformation	System size (atoms or beads)	Length
MD unbiased	apo-open	238,657	3 × 600 ns
MD unbiased	holo-open	194,959	3 × 600 ns
MD unbiased	apo-closed	284,578	3 × 600 ns
MD unbiased	holo-closed	194,905	3 × 600 ns
HREMD	apo-open	238,657	30 × 100 ns
HREMD	apo-closed	284,578	30 × 60 ns
MD pulling	apo-closed to open	238,657	0.8 ns
USMD	apo	238,519 to 1,537,576	74 × 100 ns
USMD	holo	787,203 to 1,536,653	67 × 100 ns
CGMD	closed monomer	64,894	1 μs
CGMD	closed monomer on membrane	51,130	1 μs
CGMD	closed dimer	127,975	1 μs
CGMD	open monomer	64,897	2 μs
CGMD	open dimer	127,913	2 μs
CGMD	open monomer on membrane	37,055	2 μs
CGMD	open dimer on membrane	51,575	2 μs
Total simulation time			37.1 μs

### 5.2 Analysis of the Simulations

If not stated otherwise, all analyses were performed using GROMACS 2018 tools. For the analysis of the HREMD simulations, only the target replica at λ = 1 was used.

#### 5.2.1 Domain–Domain Analysis

To characterize the interfaces between the domains of the closed BDLP monomer we calculated the evolutionary conservation profile using *ConSurf* ^54^ as well as per-residue binding free energy using *gmx*_*MMPBSA*.^55^

#### 5.2.2 Distance and Angle Definitions

The distance *d*_GT_ was calculated between the C_α_ atoms of residues N224 and T453. The angle defining hinge 1 was measured between the C_α_ atoms of residues G124, Q359, and D587, and the angle defining hinge 2 was determined between the C_α_ atoms of residues S291, P303, and L323.

#### 5.2.3 Spatial Distribution of G580

To quantify the motions of the BDLP trunk we calculated the spatial distribution of residue 580 over the trajectory using *gmx spatial*. The resulting grid was visualized in PyMol.

#### 5.2.4 Root Mean Square Fluctuations (RMSF)

We calculated the fluctuations of the C_α_ atoms around their time-averaged positions. The resulting values (between 0.1 and 0.8 nm) were projected onto the corresponding MD starting structure, with red for flexible residues and blue for rigid ones.

#### 5.2.5 Principal Component Analysis (PCA)

To observe the largest motions within the G domain, PCA was performed on the atomic motions of the flexible parts of the G domain, namely flap 1, flap 2, hinge 2, and switch 2. To this end, the trajectory was fitted to the backbone of the G domain, and PCA was then applied to the selected structural motifs. The principal motion (corresponding to component 1) was visualized using the two conformations with maximum amplitude for each BDLP state.

#### 5.2.6 Clustering Analysis

For the clustering of the G domain motifs sampled in the MD simulations, the trajectory was first fitted to the C_α_ atoms of the G domain, with a time step of 60 ps between snapshots. Clustering was then performed for the motifs flap 1, flap 2, hinge 2, and switch 2 with a C_α_ root mean square deviation (RMSD) cutoff of 0.25 nm to define cluster membership. For clustering whole protein conformations sampled within a USMD window at a specified distance *d*_GT_, we performed a clustering with a C_α_ RMSD cutoff value of 0.2 nm. In both clustering cases, the algorithm of Daura et al.^56^ was used.

#### 5.2.7 Free Energy Surfaces (FES)

Using a custom Python script applied to the 3 × 600 ns MD data per system, the free energy Δ*G* was calculated along two selected order parameters *r*_1_ and *r*_2_ using the equation

1 $$\Delta G\left(r_{1},r_{2}\right)=k_{B}T\left[\ln P\left(r_{1},r_{2}\right)-\ln P_{\max}\left(r_{1},r_{2}\right)\right]$$

Here, *k*_B_ is the Boltzmann constant, *T* is the temperature, *P*(*r*_1_,*r*_2_) is the probability of the protein adopting the two order parameters *r*_1_ and *r*_2_, and *P*_max_(*r*_1_, *r*_2_) denotes the maximum of this probability, which is subtracted to ensure that Δ*G* = 0 for the lowest free energy minimum. For *r*_1_, the distance *d*_GT_ was chosen, which was combined with the hinge 1 angle, hinge 2 angle, or the lateral trunk motion defined as Δ*z*, which serves as *r*_2_.

#### 5.2.8 Dynamic Cross Correlation (DCC)

To identify (anti)­correlations in the atomic motions within BDLP during the MD simulations, we applied DCC analysis as available in MD-TASK.^57^ This analysis generates an *N* × *N* matrix, where *N* = 692 is the number of C_α_ atoms, and each matrix element (*i*, *j*) corresponds to the dynamical cross-correlation between atoms *i* and *j*. The matrix was plotted as a heatmap, using red colors for correlations with values between 0.1 and 1 (complete correlation), blue colors for anticorrelations with values from −0.1 to −1 (complete anticorrelation), and white for negligible (anti)­correlations with DCC values in the range of 0 ± 0.1. For projecting the DCC results onto the protein structure, we used the neck and G domain residues 0–350 and 660–692 as a reference. We calculated the average DCC value for this region and then determined the 692 ΔDCC values with respect to this average. These difference values were rescaled to the range of −1 for anticorrelations and +1 for correlations, then mapped onto the protein structures using the same blue-to-red color scale as the DCC heatmap. The DCC analysis was applied to the four 600 ns MD trajectories of BDLP (apo/holo, open/closed), with a time step of 200 ps between the considered snapshots (100 frames).

#### 5.2.9 Weighted Histogram Analysis Method (WHAM)

WHAM was utilized through GROMACS^58^ to analyze the 74 and 67 USMD windows of apo- and holo-BDLP, respectively. Each window was 100 ns long, with the temperature set at 303 K, using the distance *d*_GT_ as the collective variable. To assess convergence, the USMD windows of apo-BDLP and holo-BDLP were evaluated using error bars generated by 500-fold bootstrapping as implemented in *gmx wham*. The resulting PMF profiles were smoothed using a window size of 5 in reflective mode. To transform the free energy barriers Δ*G* ^#^ for the closed-to-open transition of apo- and holo-BDLP, which are derived from the PMF profiles, into time scales for this motion, we applied the Arrhenius equation

2 $$\tau =\tau_{0}\exp\left(\frac{\Delta G^{\#}}{k_{B}T}\right)$$

Here, τ_0_ ≈ 10^–12^ s is the pre-exponential factor or Arrhenius factor, and at 303 K the thermal energy *k*_B_ *T* ≈ 0.602 kcal/mol.

#### 5.2.10 Figure and Plot Generation

The figures were generated with Python, PyMOL, Inkscape, and BioRender.

## Supplementary Material



## Data Availability

All data that support the findings of this study are included in this manuscript and in the Supporting Information. All simulation run input files, comprising the starting configuration and all necessary parameters for performing the MD simulations, are deposited at https://figshare.com/s/c81e33ebb79a98214112. The MD data were generated using GROMACS versions 2018.6 and 2020.2, with some simulations conducted in conjunction with the PLUMED plugin (version 2.4.1). For some of the MD setup generation, CHARMM-GUI and ClusPro were utilized. All trajectories were analyzed using GROMACS tools, Python, and MD-TASK.

## Notes

Methods are provided within supporting section 1; structures and parametrization (subsections: 1.1); simulation protocol for the all-atom MD simulations (subsections: 1.2); details for the HREMD and USMD simulations (subsections: 1.3), and simulation protocol for the coarse-grained MD simulations (subsections: 1.4); the additional figures in supporting section 2 comprise: protein conservation scores and MM/PBSA analysis of BDLP (Figure S1); RMSF analysis of BDLP during triplicate 600 ns all-atom MD simulations (Figure S2); snapshots of different hinge 2 motions (Figure S3); spatial distribution of the BDLP stalk tip in the apo- and holo-states (Figure S4); quantification of hinge 1 motion in the apo- and holo-states of BDLP (Figure S5); evolution of the secondary structure of BDLP during all-atom MD simulations (Figure S6); dynamic cross correlation between residue motions in all-atom MD simulations (Figure S7); and umbrella sampling MD simulations of BDLP (Figure S8) (PDF)
