# Supplementary material for: From Closed to Mighty: How GTP-Induced Allosteric Dynamics, Dimerization, and Membrane Binding Transform a Bacterial Dynamin-like Protein
Source: J Chem Inf Model. 2026 Sep 23;66(19):12862–76. doi: 10.1021/acs.jcim.6c01952 (PMC13663481; doi:10.1021/acs.jcim.6c01952)
Supplement: Supplementary file 1 [file ci6c01952_si_001.pdf]

Supplementary Information: From Closed to Mighty: How GTP-Induced Allosteric Dynamics, Dimerization, and Membrane Binding Transform a Bacterial Dynamin-Like Protein

Wibke Schumann,[†] Bastian F. Bundschuh,[†,‡] Jennifer Loschwitz,[†] and Birgit Strodel[*,†,‡]

†*Faculty of Mathematics and Natural Sciences, Heinrich Heine University Düsseldorf, 40225 Düsseldorf, Germany*

‡*Institute of Biological Information Processing: Structural Biochemistry, Forschungszentrum Jülich, 52428 Jülich, Germany*

E-mail: b.strodel@fz-juelich.de

1 Supplementary methods

1.1 Structures and parametrization

The structures of BDLP were taken from the Protein Data Bank (PDB 2J68 and 2W6D)[1,2] and modeled using RCD+, GalaxyLoop, DaReUs-Loop, and ModLoop.[3–6] For the MD simulations, the Amber99SB*-ILDNP force field(,[7–9] TIP3P water[10] and GROMACS (version 2018.6)[11] were used, at a temperature of 303 K (30°C) and a pressure of 1 bar. A newer version of GROMACS was not used because we aimed to employ the same GROMACS version for both standard and enhanced MD simulations (as detailed below). We only had a functioning GROMACS-PLUMED setup for version 2018.6 of GROMACS. GTP was parameterized following the protocol by Loschwitz et al.[12] and docked into BDLP using AutoDock.[13] The GDP-bound crystal structure (PDB 2J68) served as a reference to position both GTP and Mg^{2+} within the binding pocket. Neither ligand was restrained during the subsequent all-atom MD, HREMD, and USMD simulations. To neutralize the system, eight Na^+ ions were added to the apo-protein solvent, and ten Na^+ ions were added to the holo-protein solvent.

1.2 Simulation protocol for the all-atom MD simulations

First, an energy minimization was performed using a steepest descent algorithm, followed by a subsequent 0.1 ns NVT equilibration and a 1 ns NpT equilibration. During these steps, the protein's heavy atoms were restrained with a force constant of 1000 kJ/(mol·nm^2). The velocity rescaling thermostat was employed to regulate the temperature in the NVT simulations, while the Nosé-Hoover thermostat[14,15] and the isotropic Parrinello-Rahman barostat[16] were used for the NpT simulations. The particle mesh-Ewald (PME)[17,18] sum method was applied to calculate electrostatic interactions with xyz-periodic boundary conditions. The van der Waals (vdW) and short-range Coulombic interaction cutoffs were set to 1.2 nm and repulsive Lennard-Jones (LJ) interactions were cut at 1.0 nm. The equations of motion were integrated using the leapfrog method, and the LINCS algorithm[19] was used to constrain all bonds. For

the production runs, the same setup as in the NpT equilibration was used, except with most position restraints removed.

In the apo-BDLP systems, selected hydrogen atoms were treated as virtual interaction sites, permitting an integration time step of 4 fs while maintaining energy conservation.[20] For the holo-BDLP systems, a time step of 2 fs was used. Coordinates and velocities were recorded every 20 ps in both cases. In all simulations, we applied position restraints to the rigid C_α atoms of the G domain β-sheets to remove overall translation and rotation, allowing us to decrease the box size without harming the protein's flexibilities.[21] All other atoms were unrestrained in the production runs.

1.3 Details for the HREMD and USMD simulations

We conducted Hamiltonian replica exchange MD (HREMD) simulations[22] of apo-BDLP with 30 replicas, running for 100 ns for the open conformation and 60 ns for the closed conformation per replica. The energy function of BDLP and its protein–water interactions were modified in each but the target replica by applying biasing factors of 310 K/T, with the 30 temperatures T exponentially distributed between 310 and 370 K ($1 < \lambda < 0.667$). The unbiased target replica at 310 K was used for analysis. The average exchange probability between the replicas was ca. 30%. The HREMD simulations were conducted with Gromacs 2018.6 patched with the PLUMED plugin (version 2.4.1).[23] In all HREMD simulations, we used the v-rescale thermostat with canonical sampling and the Parrinello-Rahman barostat.[16] Apart from that, the setup was identical to the regular production runs described above.

From the unrestrained simulations, the predominant motion was identified using principal component analysis. The motion was then described using a distance, measured between the C_α atoms of residues 224 and 453. This order parameter/reaction coordinate is referred to as d_{GT} and used for the USMD simulations. To generate the intermediate states between the open and closed form of BDLP, a 0.8 ns pulling simulations via the pulling code in GROMACS with 1000 kJ/(mol·nm^2) force constant. In the USMD, 100 apo-BDLP conformations along

d_{GT} were simulated for 100 ns each. The force constants for restraining the conformations along d_{GT} ranged between 3 and 6,200 kJ/(mol·nm^2), depending on the stability of the different windows; though the majority of the windows were simulated with 500 kJ/(mol·nm^2). This setup was repeated for holo-BDLP. Of the 100 windows, only those which stayed in the range of the original window were used for analysis, resulting in 74 and 67 windows for apo-BDLP and holo-BDLP, respectively. The free energy profile from the USMD simulations was obtained from a WHAM analysis.[24,25]

1.4 Simulation protocol for the coarse-grained MD simulations

The CG simulations were realized with the MARTINI 2.2 force field[26] and GROMACS (version 2020.2), using CHARMM-GUI[27] for generating the setup of these simulations. The output from CHARMM-GUI was then modified using the domELNEDIN script[28] to ensure that the protein was restrained by elastic networks, but only within domains. As MARTINI 2 is known to overstabilize protein–protein interactions,[29] we conducted test simulations of the BDLP dimers using the MARTINI 3 force field,[30] which rectified this issue, along with the elastic network ELNEDIN[31] to preserve the overall shape of the individual proteins. Since the dimers did not dissociate within 1 μs during these MARTINI 3 simulations, we concluded that it is safe to apply MARTINI 2.2, which allows for modeling interdomain flexibility when used in conjunction with domELNEDIN. The dimer structures were generated through protein–protein docking using ClusPro,[32] with selecting the predictions based on their similarity to the known crystal structure of the open form (PDB 2W6D) and the closed dimer described in the reference.[1] In the absence of knowledge about *N.punctiforme* membrane composition, POPC was used as a generalized membrane model.

To avoid potential artifacts associated with modeling highly charged coordination complexes at a coarse-grained resolution, GTP and Mg^{2+} were not explicitly resolved in the Martini 2.2 simulations but were implicitly considered by restraining the G-domain to the holo-state geometry. This approach was previously validated in our prior work.[21] The energy

minimization, equilibration, and production simulations were conducted according to the .mdp files generated by CHARMM-GUI. Following production simulations at 303 K and 1 bar (1 μs for closed and 2 μs for open BDLP structures), backmapping of the final MD snapshot for each system to the atomistic level was performed using the CHARMM-GUI all-atom converter, followed by energy minimization in vacuum.

2 Supplementary figures

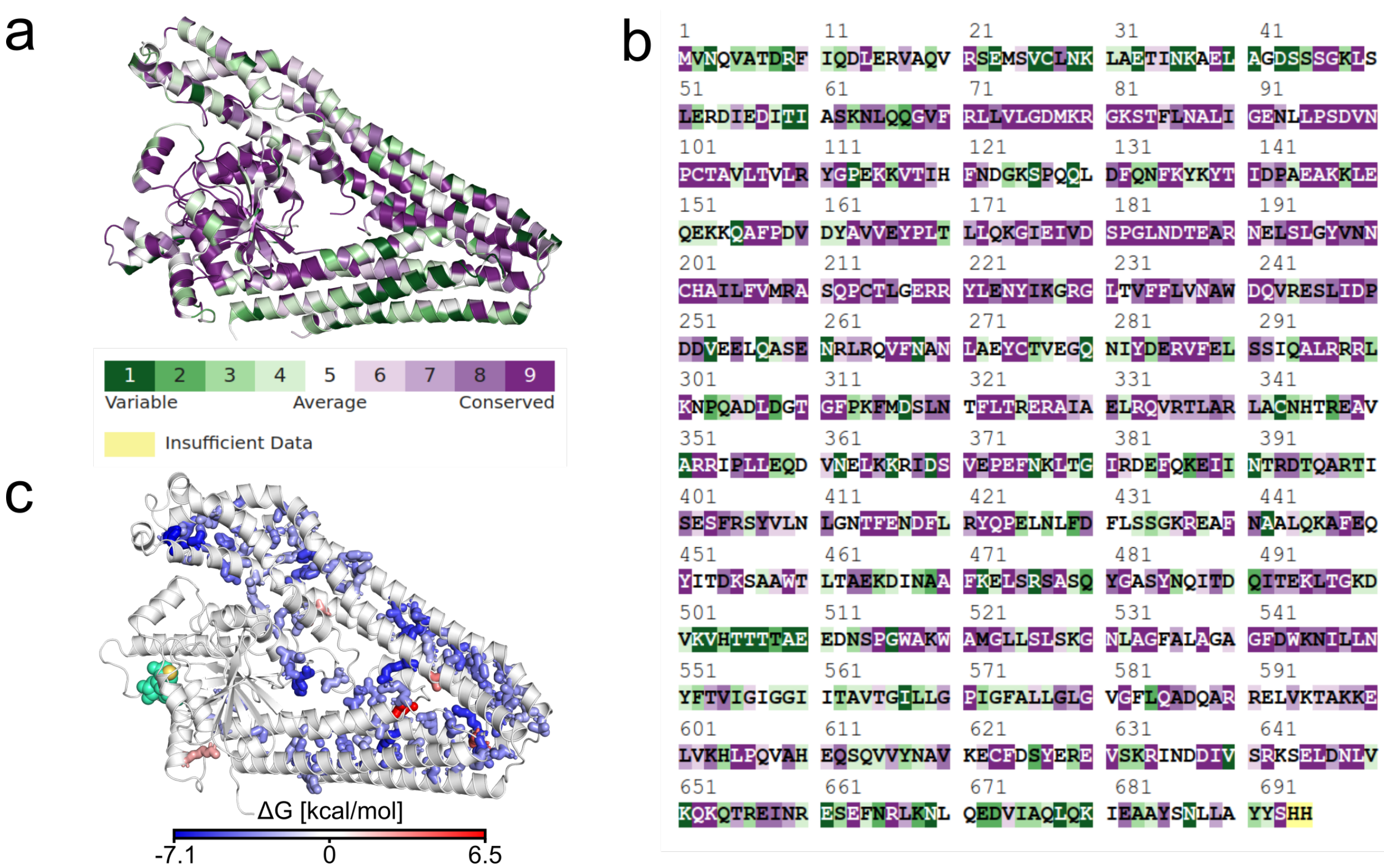

Figure S1: Protein conservation scores and MM/PBSA analysis of BDLP. (**a**) Protein conservation scores calculated by the ConSurf server mapped onto the BDLP structure and (**b**) its amino acid sequence, using a nine-grade color scale ranging from green (variable) to purple (conserved). Residues for which insufficient data precluded analysis are shown in yellow. (**c**) Per-residue binding free energies obtained from MM/PBSA analysis of the closed BDLP conformation mapped onto the structure, using a color spectrum from blue (favorable/negative) through white (neutral) to red (unfavorable/positive). Energies were calculated exclusively for interdomain contacts.

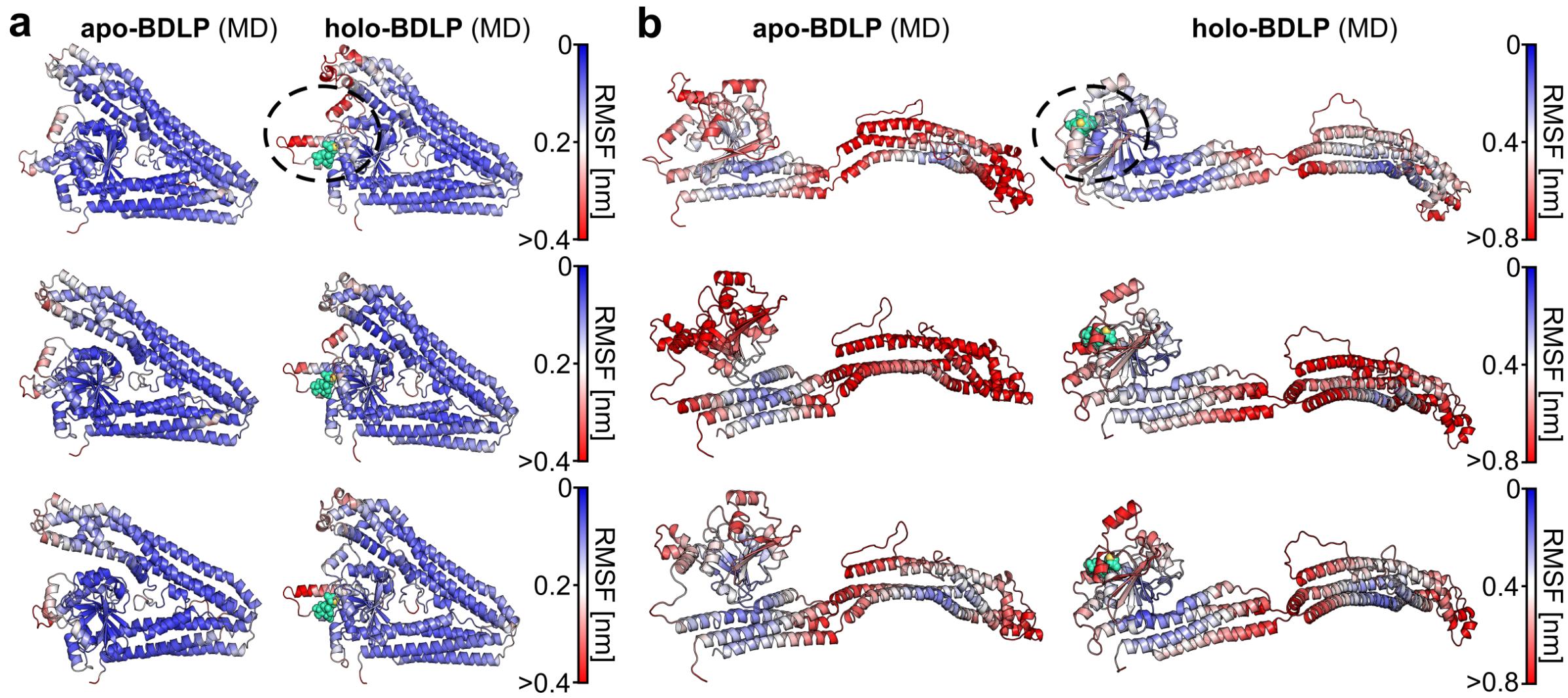

Figure S2: RMSF analysis of BDLP during triplicate 600 ns all-atom MD simulations. (**a**) RMSF values for the closed BDLP conformation mapped onto the structures for the apo- (left) and holo-protein (right), with replicates arranged from top to bottom. The color scale ranges from blue for low RMSF (≈ 0 nm) to red for high RMSF (≥ 0.4 nm). Regions of elevated flexibility in holo-BDLP are observed in the structural elements near the GTP-binding site (flap 1 and flap 2) and at the G-domain/paddle interface (indicated by a dashed black circle). (**b**) RMSF values for the open BDLP conformation mapped onto the structures for the apo- (left) and holo-protein (right), with replicates arranged from top to bottom. The color scale ranges from blue for low RMSF (≈ 0 nm) to red for high RMSF (≥ 0.8 nm).

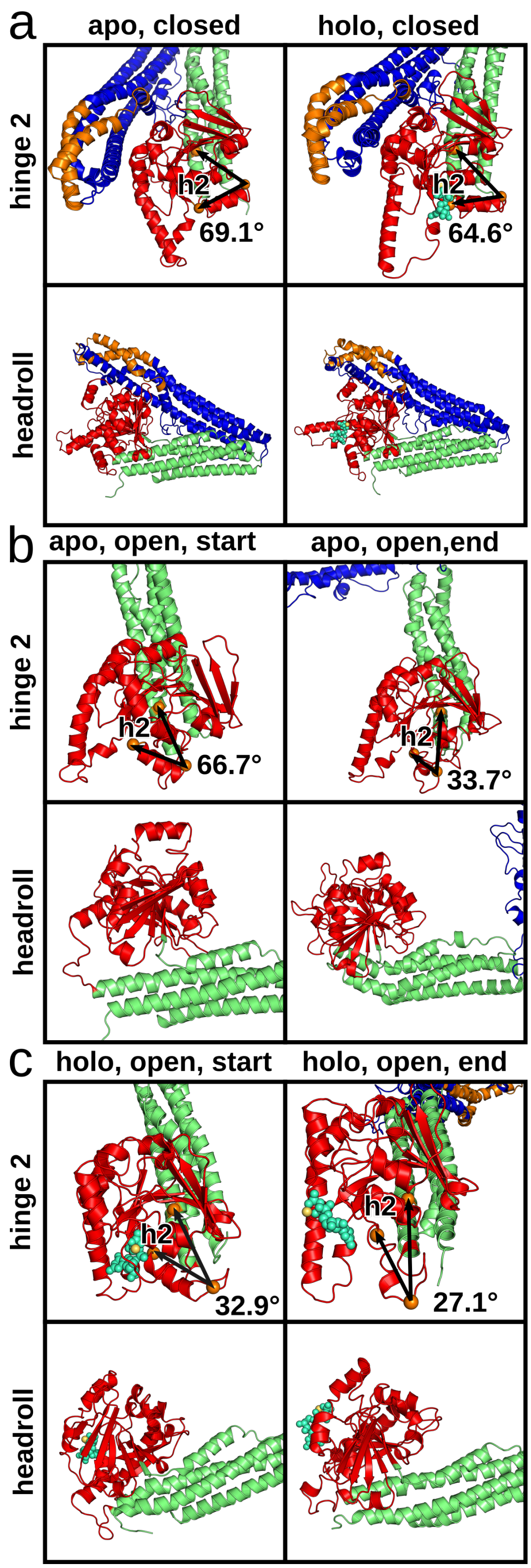

Figure S3: Snapshots of different hinge 2 motions. Hinge 2 is defined by the angle between S291, P303, and L323, which are indicated by brown spheres in the upper row per panel a, b, and c. In the lower row per panel, the corresponding position of the GTPase domain with respect to the neck region is shown.

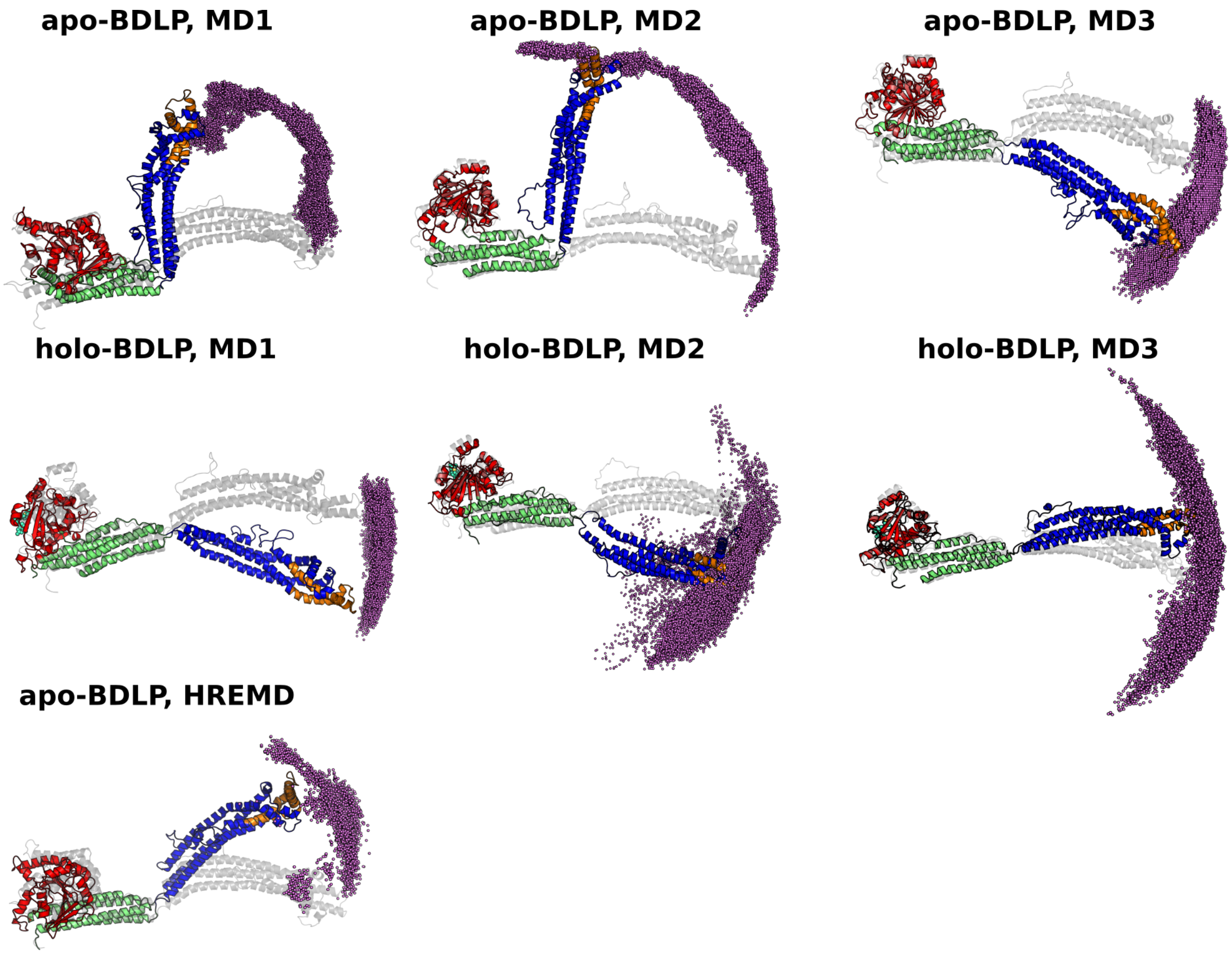

Figure S4: Spatial distribution of the BDLP stalk tip in the apo- and holo-states. The distribution of the BDLP stalk tip (G580) is projected as purple points sampled during the triplicate all-atom MD simulations of the apo- and holo-protein, as well as within the target replica of the apo-BDLP monomer HREMD simulation. The open starting conformation is displayed in a gray cartoon representation for reference.

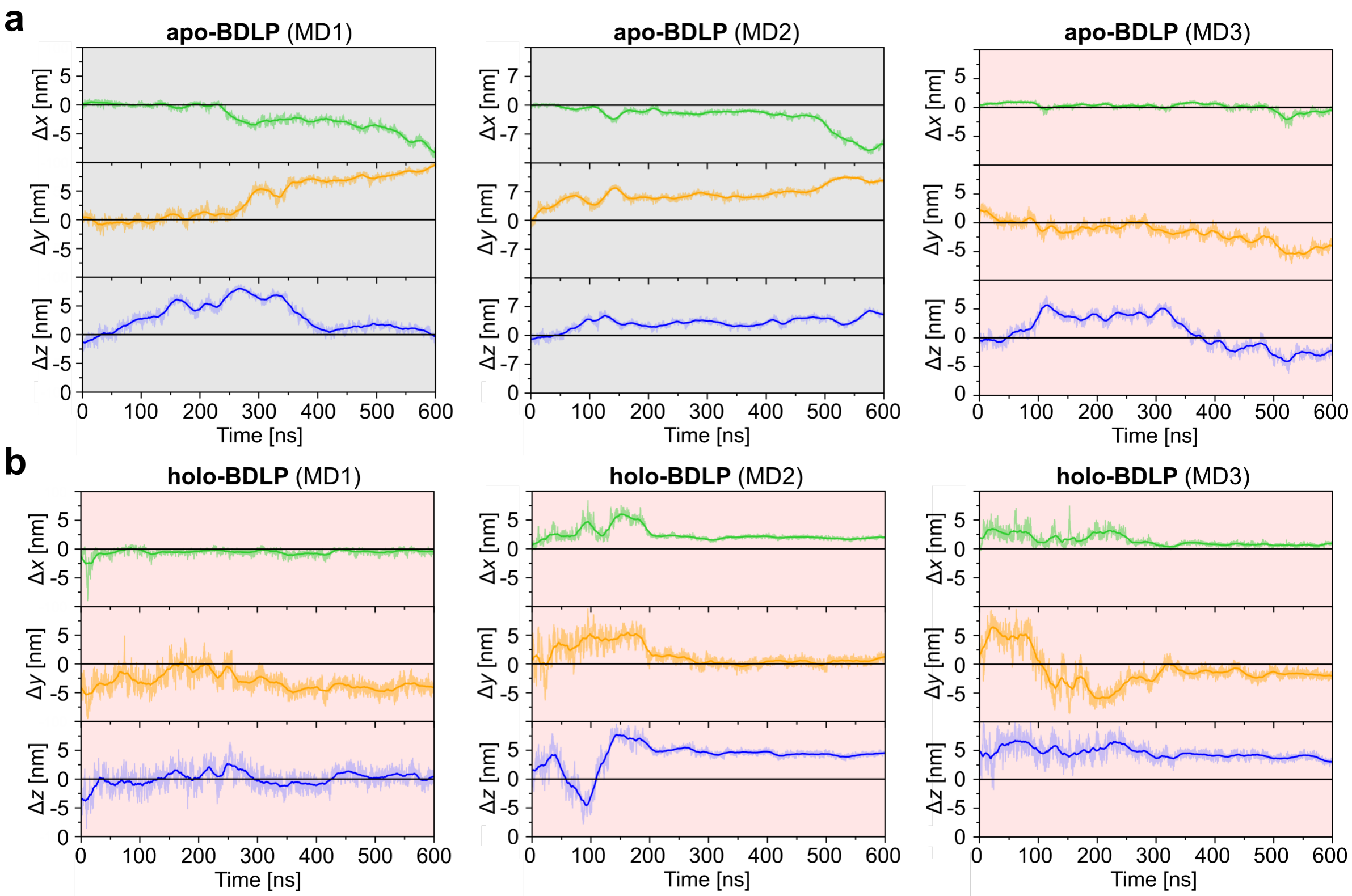

Figure S5: Quantification of hinge 1 motion in the apo- and holo-states of BDLP. The hinge 1 motion is characterized by the Cartesian displacement of the stalk tip (represented by residue G580), using the coordinate system defined in Fig. 2c. Phases of closing and opening associated with the hinge 1 dynamics are indicated by gray and pink backgrounds, respectively. Results from the triplicate all-atom MD runs (arranged left to right) are shown for the open conformation of (**a**) apo-BDLP and (**b**) holo-BDLP.

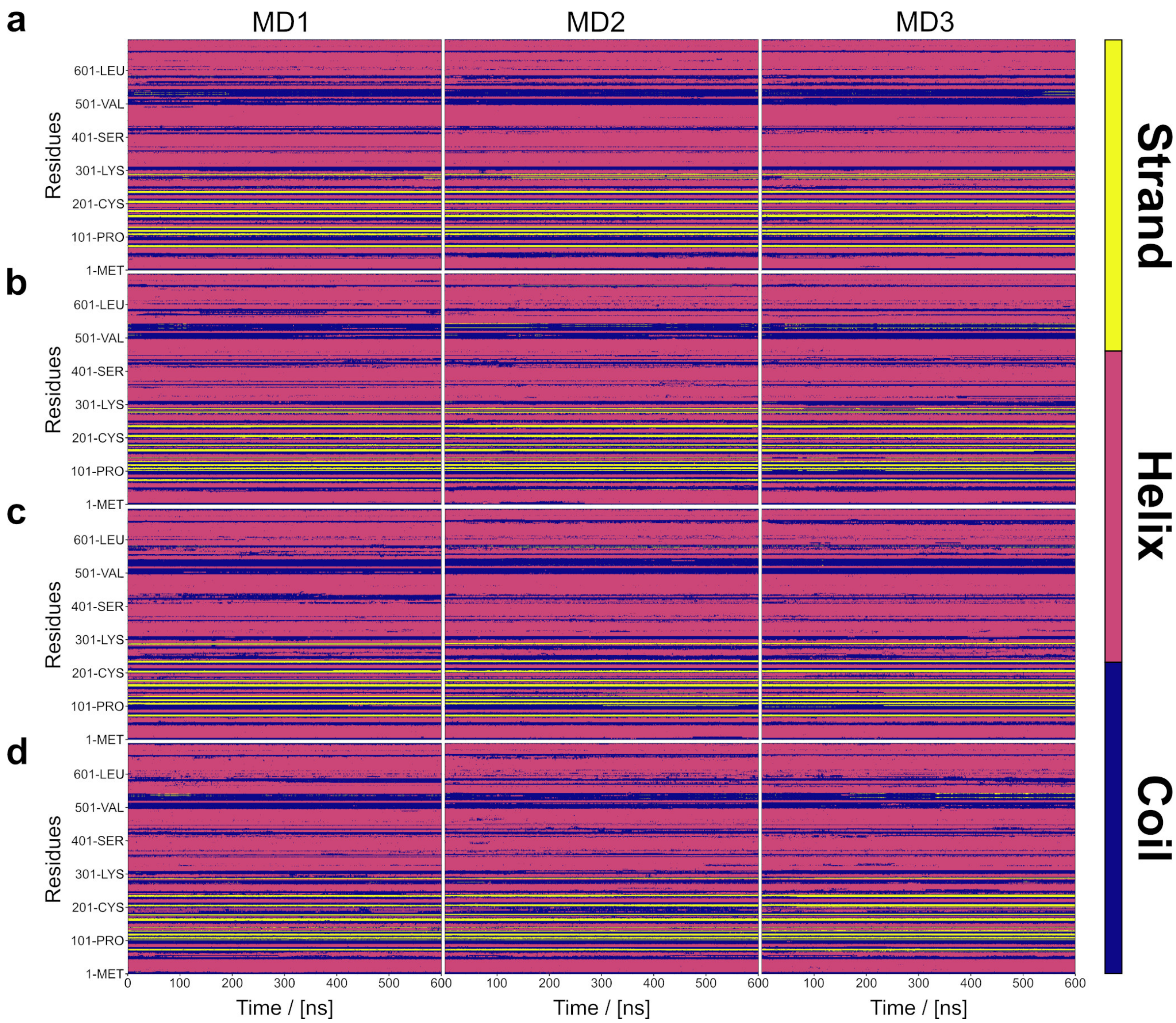

Figure S6: Evolution of the secondary structure of BDLP during all-atom MD simulations. The secondary structure per residue (determined via DSSP) is shown for the triplicate MD runs (arranged left to right) of (**a**) closed apo-BDLP, (**b**) open apo-BDLP, (**c**) closed holo-BDLP, and (**d**) open holo-BDLP. Structural elements are classified into β-strands (yellow), α-helices (pink), and turns/random coils (blue). The profiles exhibit minimal secondary structure alterations, reflecting high stability at this structural level despite large-scale hinge 1 motions.

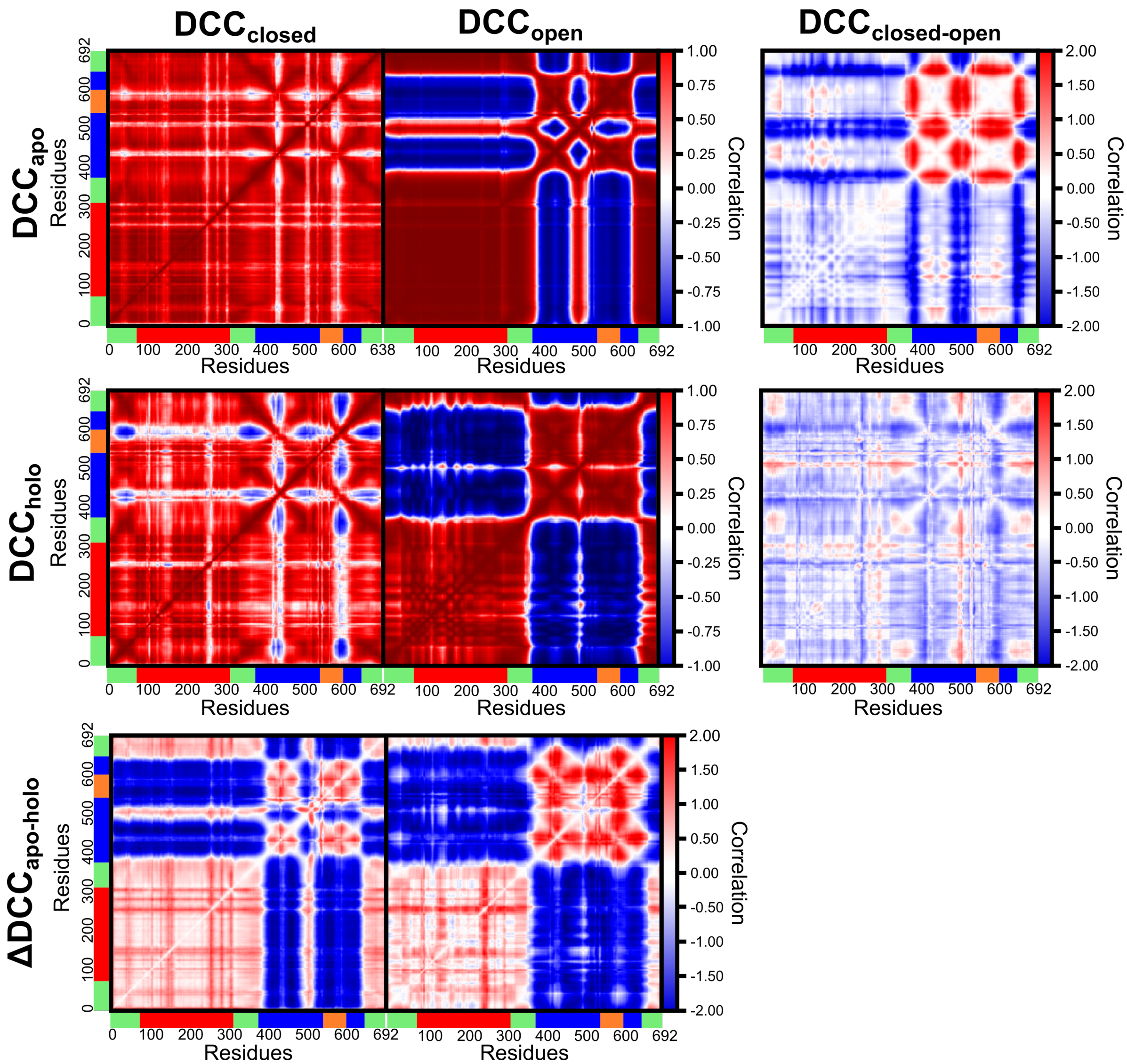

Figure S7: Dynamic cross correlation between residue motions in all-atom MD simulations. The DCC maps of the closed (top left) and open forms (middle left) in both apo- and holo-states, as well as the differences $\Delta\mathrm{DCC}_{\mathrm{apo-holo}}$ (bottom left) and $\Delta\mathrm{DCC}_{\mathrm{closed-open}}$ (top and middle right), are displayed. The DCC values are represented as heatmaps, with red colors indicating correlation values between +0.1 and +1 (correlation) and blue colors representing values from −0.1 to −1 (anti-correlation). The color bar for the DCC difference plots ranges from −2 (anti-correlation, blue colors) to +2 (correlation, red colors). The axes labels show the residue numbers along with a color code that assigns the residues to the domains as introduced in Fig. 1.

.

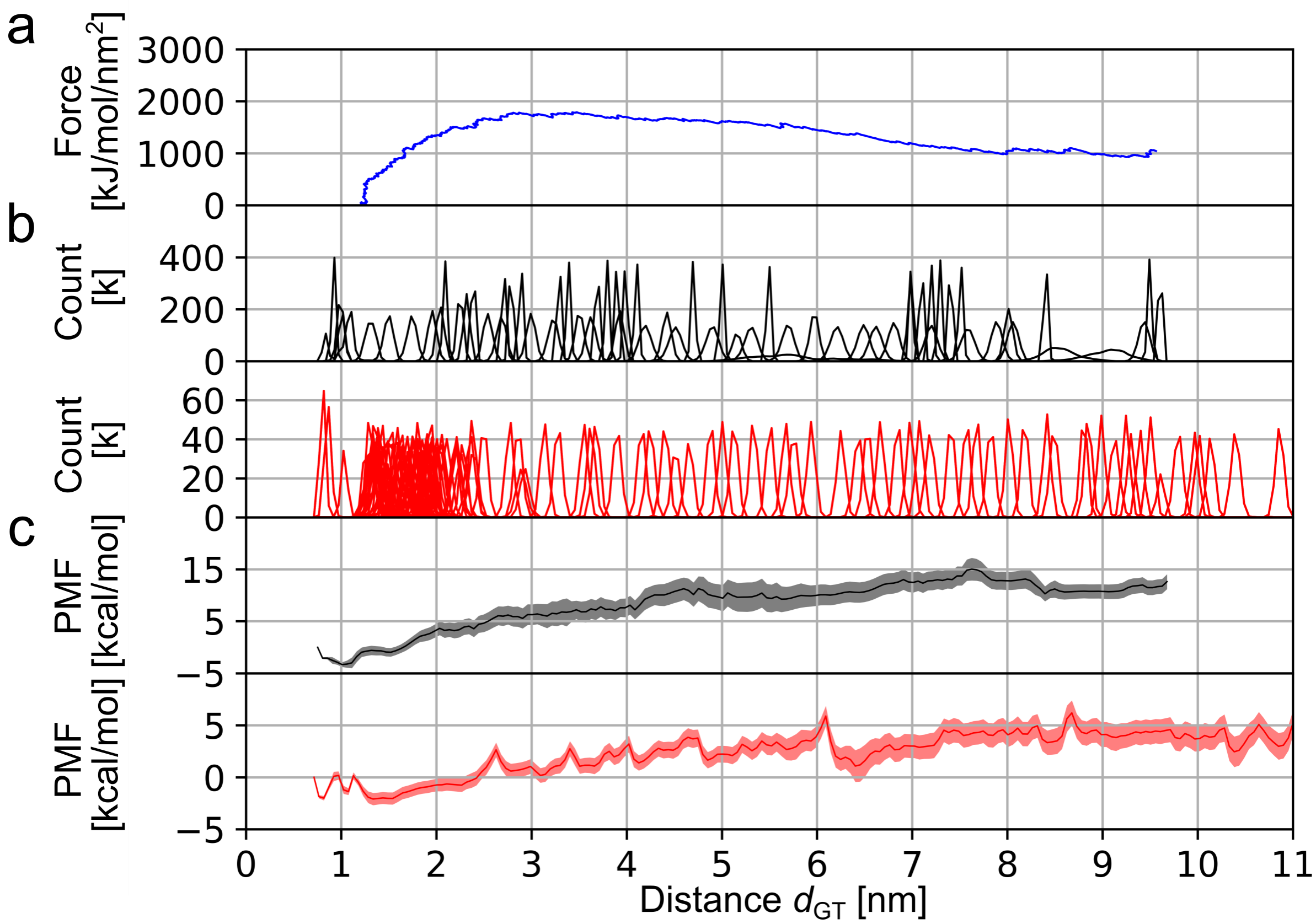

Figure S8: Umbrella sampling MD simulations of BDLP. (**a**) The force profile from the initial pulling simulation of apo-BDLP is shown. (**b**) Coverage of the order parameter d_{GT} by the windows in the USMD simulations of apo-BDLP (black) and holo-BDLP (red) are depicted. (**c**) Convergence test for the USMD simulation of apo-BDLP (black) and holo-BDLP (red) using 500-fold bootstrapping; the standard error is represented as shaded area.

References

(1) Low, H. H.; Löwe, J. A bacterial dynamin-like protein. *Nature* **2006**, *444*, 766–769.

(2) Low, H. H.; Löwe, J. Dynamin architecture — from monomer to polymer. *Curr. Opin. Struct. Biol.* **2010**, *20*, 791–798.

(3) Park, H.; Lee, G. R.; Heo, L.; Seok, C. Protein loop modeling using a new hybrid energy function and its application to modeling in inaccurate structural environments. *PLoS ONE* **2014**, *9*.

(4) López-Blanco, J. R.; Canosa-Valls, A. J.; Li, Y.; Chacón, P. RCD+: Fast loop modeling server. *Nucleic Acids Res.* **2016**, *44*, W395–W400.

(5) Karami, Y.; Rey, J.; Postic, G.; Murail, S.; Tufféry, P.; de Vries, S. J. DaReUS-Loop: a web server to model multiple loops in homology models. *Nucleic Acids Res.* **2019**, *47*, W423–W428.

(6) Fiser, A.; Sali, A. ModLoop: automated modeling of loops in protein structures. *Bioinformatics* **2003**, *19*, 2500–2501.

(7) Lindorff-Larsen, K.; Piana, S.; Palmo, K.; Maragakis, P.; Klepeis, J. L.; Dror, R. O.; Shaw, D. E. Improved side-chain torsion potentials for the Amber ff99SB protein force field. *Proteins* **2010**, *78*, 1950–1958.

(8) Best, R. B.; Hummer, G. Optimized molecular dynamics force fields applied to the helix-coil transition of polypeptides. *J. Phys. Chem. B* **2009**, *113*, 9004.

(9) Aliev, A. E.; Kulke, M.; Khaneja, H. S.; Chudasama, V.; Sheppard, T. D.; Lanigan, R. M. Motional timescale predictions by molecular dynamics simulations: Case study using proline and hydroxyproline sidechain dynamics. *Proteins* **2014**, *82*, 195.

(10) Jorgensen, W. L.; Chandrasekhar, J.; Madura, J. D.; Impey, R. W.; Klein, M. L. Comparison of simple potential functions for simulating liquid water. *J. Chem. Phys.* **1998**, *79*, 926.

(11) Abraham, M. J.; Murtola, T.; Schulz, R.; Páll, S.; Smith, J. C.; Hess, B.; Lindahl, E. GROMACS: High performance molecular simulations through multi-level parallelism from laptops to supercomputers. *SoftwareX* **2015**, *1-2*, 19–25.

(12) Loschwitz, J.; Steffens, N.; Wang, X.; Schäffler, M.; Pfeffer, K.; Degrandi, D.; Strodel, B. Domain motions, dimerization, and membrane interactions of the murine guanylate binding protein 2. *Sci. Rep.* **2023**, *13*, 679.

(13) Trott, O.; Olson, A. J. AutoDock Vina: Improving the speed and accuracy of docking with a new scoring function, efficient optimization, and multithreading. *J. Comput.*

Chem. **2009**, *31*.

(14) Hoover, W. G. Canonical dynamics: Equilibrium phase-space distributions. *Phys. Rev. A* **1985**, *31*, 1695.

(15) Nosé, S. A molecular dynamics method for simulations in the canonical ensemble. *Mol. Phys.* **1984**, *52*, 255–268.

(16) Parrinello, M.; Rahman, A. Polymorphic transitions in single crystals: A new molecular dynamics method. *J. Appl. Phys.* **1998**, *52*, 7182.

(17) T. Darden, D. Y.; Pedersen, L. PARTICLE Mesh Ewald - an N.Log(N) Method for Ewald Sums in Large Systems. *J. Chem. Phys.* **1993**, *98*, 10089–10092.

(18) Essmann, U.; Perera, L.; Berkowitz, M. L. A Smooth PARTICLE Mesh Ewald Method. *J. Chem. Phys.* **1995**, *103*, 8577–8593.

(19) Hess, B.; Bekker, H.; Berendsen, H. J. C.; Fraaije, J. LINCS: A linear constraint solver for molecular simulations. *J. Comput. Chem.* **1997**, *18*, 1463–1472.

(20) Feenstra, K. A.; Hess, B.; Berendsen, H. J. C. Improving efficiency of large time-scale molecular dynamics simulations of hydrogen-rich systems. *J. Comput. Chem.* **1999**, *20*, 786–798.

(21) Barz, B.; Loschwitz, J.; Strodel, B. Large-scale, dynamin-like motions of the human guanylate binding protein 1 revealed by multi-resolution simulations. *PLoS Comput. Biol.* **2019**, *15*, e1007193.

(22) Bussi, G. Hamiltonian replica exchange in GROMACS: a flexible implementation. *Mol. Phys.* **2014**, *112*, 379–384.

(23) Tribello, G. A.; Bonomi, M.; Branduardi, D.; Camilloni, C.; Bussi, G. PLUMED 2: New feathers for an old bird. *Comp. Phys. Commun.* **2014**, *185*, 604–613.

(24) Hub, J. S.; de Groot, B. L.; van der Spoel, D. g_wham - A Free Weighted Histogram Analysis Implementation Including Robust Error and Autocorrelation Estimates. *J. Chem. Theory Comput.* **2010**, *6*, 3713–3720.

(25) Kästner, J. Umbrella sampling. *Wiley Interdiscip. Rev.-Comput. Mol. Sci.* **2011**, *1*, 932–942.

(26) de Jong, D. H.; Singh, G.; Bennett, W. F. D.; Arnarez, C.; Wassenaar, T. A.; Schäfer, L. V.; Periole, X.; Tieleman, D. P.; Marrink, S. J. Improved Parameters for the Martini Coarse-Grained Protein Force Field. *J. Chem. Theory Comput.* **2012**, *9*, 687–697.

(27) Jo, S.; Kim, T.; Iyer, V. G.; Im, W. CHARMM-GUI: A web-based graphical user interface for CHARMM. *J. Comput. Chem.* **2008**, *29*, 1859–1865.

(28) Siuda, I.; Thøgersen, L. Conformational flexibility of the leucine binding protein examined by protein domain coarse-grained molecular dynamics. *J. Mol. Model.* **2013**, *19*, 4931–4945.

(29) Javanainen, M.; Martinez-Seara, H.; Vattulainen, I. Excessive aggregation of membrane proteins in the Martini model. *PLOS ONE* **2017**, *12*, e0187936.

(30) Souza, P. C. T. et al. Martini 3: a general purpose force field for coarse-grained molecular dynamics. *Nature Meth.* **2021**, *18*, 382–388.

(31) Periole, X.; Cavalli, M.; Marrink, S.-J.; Ceruso, M. A. Combining an Elastic Network With a Coarse-Grained Molecular Force Field: Structure, Dynamics, and Intermolecular Recognition. *J. Chem. Theory Comput.* **2009**, *5*, 2531–2543.

(32) Kozakov, D.; Hall, D. R.; Xia, B.; Porter, K. A.; Padhorny, D.; Yueh, C.; Beglov, D.; Vajda, S. The ClusPro web server for protein–protein docking. *Nat. Protoc.* **2017**, *12*, 255–278.
